# Distinct mechanisms orchestrate the contra-polarity of IRK and KOIN, two LRR-receptor-kinases controlling root cell division

**DOI:** 10.1038/s41467-021-27913-1

**Published:** 2022-01-11

**Authors:** Cecilia Rodriguez-Furlan, Roya Campos, Jessica N. Toth, Jaimie M. Van Norman

**Affiliations:** Department of Botany and Plant Sciences, Center for Plant Cell Biology, Institute of Integrative Genome Biology, University of California, Riverside, Riverside, CA 92521 USA

**Keywords:** Plant polarity, Root apical meristem, Cell polarity

## Abstract

In plants, cell polarity plays key roles in coordinating developmental processes. Despite the characterization of several polarly localized plasma membrane proteins, the mechanisms connecting protein dynamics with cellular functions often remain unclear. Here, we introduce a polarized receptor, KOIN, that restricts cell divisions in the Arabidopsis root meristem. In the endodermis, KOIN polarity is opposite to IRK, a receptor that represses endodermal cell divisions. Their contra-polar localization facilitates dissection of polarity mechanisms and the links between polarity and function. We find that IRK and KOIN are recognized, sorted, and secreted through distinct pathways. IRK extracellular domains determine its polarity and partially rescue the mutant phenotype, whereas KOIN’s extracellular domains are insufficient for polar sorting and function. Endodermal expression of an IRK/KOIN chimera generates non-cell-autonomous misregulation of root cell divisions that impacts patterning. Altogether, we reveal two contrasting mechanisms determining these receptors’ polarity and link their polarity to cell divisions in root tissue patterning.

## Abstract (web summary)

Protein polarization coordinates many plant developmental processes. Here the authors show that IRK and KOIN, two LRR-receptor-kinases polarized to opposite sides of cells in the root meristem, rely on distinct mechanisms to achieve polarity.

## Introduction

Asymmetric protein distribution along different plasma membrane (PM) domains can be guided by external cues from adjacent or distal cells and may define polar axes of a tissue or organ. This coordinated polarity facilitates oriented cell division, developmental patterning, cell growth, long-range signal transduction, and the transport of ions and other molecules^1^. Intrinsic cellular machinery generates polar protein distribution, but the process remains poorly understood^2,3^. However, research has been greatly aided by examining PM proteins with contrasting polar distribution^1^.

A well-studied example of proteins with asymmetric distribution at the PM is the PIN-FORMED (PIN) family of auxin efflux carriers that drive directional auxin flow, which is essential for root growth and development. When expressed under their endogenous promoters, PIN1 and PIN2 exhibit contrasting polar distribution. PIN1 accumulates at the rootward PM domain in vascular and endodermal cells, while PIN2 is located at the shootward domain in epidermal and cortex cells. PIN1 polar distribution changes in different cell types indicating cell type-specific localization mechanisms^4^. PIN2 rootward localization in cortex cells near the stem cell niche becomes shootward as cells mature, indicating its polarity is associated with developmental context^4^. PIN polarity is established after cytokinesis through distinct trafficking routes for shootward/rootward localization^4–7^. Once established, maintenance of PIN polarity occurs by hyper-polar secretion, endocytosis and recycling, restriction of lateral diffusion, and protein phosphorylation^8^. However, the sorting signals and regulatory mechanisms targeting these proteins in different cell types and developmental contexts are still being investigated.

Another well-studied, contrasting protein duo is the laterally localized boric acid channel NODULIN INTRINSIC PROTEIN 5;1 (NIP5;1) and the boric acid/borate exporter REQUIRES HIGH BORON 1 (BOR1)^9^. Contra-polar localization of BOR1 at the inner PM domain toward the stele and NIP5;1 at the outer domain away from the stele, is proposed to be determined by cues originating from the stele^10^. This polar distribution is maintained in different tissues indicating an organ-level, stele-oriented polar axis enabling directional boron transport. Under boron limiting conditions, BOR1 and NIP5;1 polar distribution in epidermal and cortical cells is regulated by targeted secretion of newly synthesized proteins, constant endocytosis and recycling, cytoskeleton components, and control of lateral diffusion^3,9,11–14^. However, in endodermal cells, once BOR1 and NIP5;1 are polarly secreted, maintenance of lateral polarity is independent of the actin or microtubule cytoskeleton^10^. Additionally, the polarity of NIP5;1, but not BOR1, depends on phosphoinsitol synthesis^10^. This suggests differential mechanisms for protein accumulation to the inner or outer PM domains in endodermal cells, yet these underlying mechanisms remain poorly understood.

The leucine-rich repeat receptor-like kinase (LRR-RLK) INFLORESCENCE AND ROOT APICES RECEPTOR KINASE (IRK) exhibits polar accumulation to distinct PM domains in different cell types^15^. It localizes to the outer PM domain in the endodermis and pericycle and the inner domain in epidermal and cortical cells, with its polar distribution informed locally by adjacent cells. IRK is downstream of SHORT ROOT (SHR) transcriptional regulation and SHR together with the transcription factor SCARECROW (SCR) control expression of a D-type cyclin, *CYCD6;1*, restricting it to the cortex/endodermal initial (CEI) and the CEI daughter (CEID) leading to formative cell divisions that eventually generate endodermal and cortical cells. Strict regulation of gene expression in these cell types prevents the formation of additional ground tissue (GT) layers and maintains radial patterning^16–20^. *CYCD6;1* promoter activity is altered in *irk* endodermal cells, which exhibit excess periclinal and longitudinal anticlinal cell divisions (LADs), leading to radial enlargement of the root. These phenotypes are rescued by endodermal IRK expression suggesting its polar distribution is functionally linked to repression of cell division in these cells upon a perception of extracellular cues^15^. However, the mechanistic basis for the establishment and maintenance of IRK polarity remains unknown.

Here, we introduce another LRR-RLK downstream of the SHR transcriptional network that, in contrast to IRK, is polarized to the inner PM domain regardless of cell type; therefore, we named it KINASE ON THE INSIDE (KOIN). The contrasting polarization of IRK and KOIN makes them a valuable pair to investigate the underlying mechanisms of LRR-RLK polarity. Our study reveals that polarization of IRK and KOIN relies mainly on the secretion of newly synthesized protein through distinct endomembrane trafficking routes. IRK and KOIN differentially accumulate during endodermal cell divisions, revealing targeted secretion to newly formed PMs. Using chimeric proteins and truncations, we found the protein domains necessary for IRK polar sorting are distinct from those of KOIN. Our results reveal coordination between LRR-RLK polarity and function to repress root cell division in the radial and longitudinal axes, adding to the growing body of work showing that polarized signal perception controls specific developmental events.

## Results

### KOIN and IRK show contrasting polar localization in the root

**Fig. 1:**
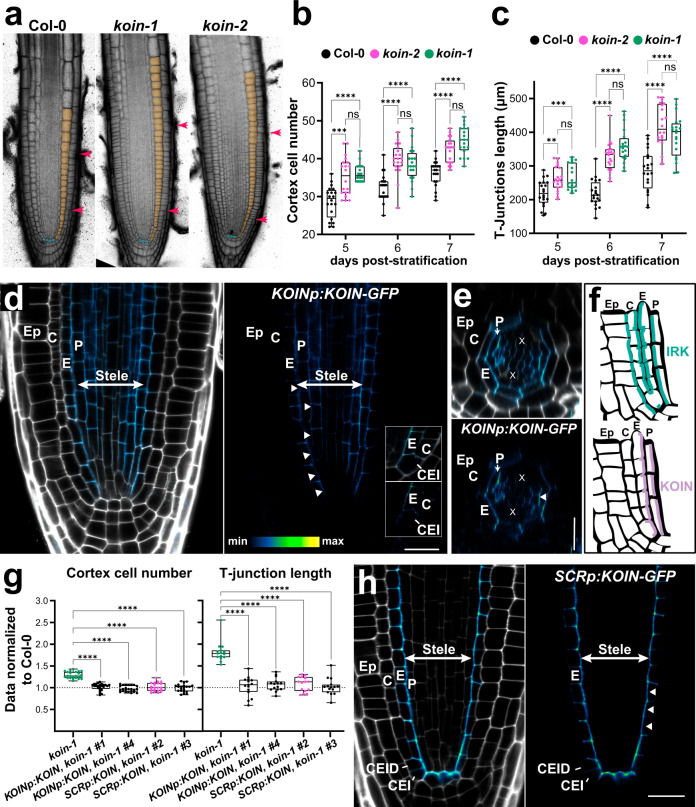
KOIN functions to restrict root meristem size and is localized to the inner lateral domain in endodermal cells. **a** Confocal images of the median longitudinal sections of root tips with cells highlighted: quiescent center (QC, cyan), meristematic cortex (orange), and T-junctions (pink arrowheads). Quantification of **b** cortex cell number and **c** distance to the uppermost T-junction over time 5–7 days post-stratification (d.p.s.). Box plots show data from a single biological replicate representative of three with similar results (*n* = 20 per genotype). Boxes delimit upper and lower quartiles, median values are indicated with a line, and single values are shown as dots. Bars indicate max/min values and **** statistical significance *P* value < 0.001 (two-way ANOVA using Turkey’s multiple comparison test). **d**, **e** Confocal images of WT root meristems at 6 d.p.s. Adjacent panels show merged images of green fluorescent protein (GFP, fluorescence intensity color scale) and stained with propidium iodide (PI, gray scale) followed by GFP alone. **d** Median longitudinal and **e** transverse sections showing accumulation of KOIN-GFP driven by *KOINp*. Inset in (**d**) KOIN-GFP accumulation in the CEI. **f** Schematics summarizing endogenously expressed IRK-GFP localization (green) and KOIN-GFP (purple). **g** Quantification of cortex cell number and distance to the uppermost T-junction at 7 d.p.s. in *koin-1* expressing KOIN-GFP driven by *KOINp* or *SCRp* normalized to Col-0. Data from *n* = 20 per genotype for a single biological replicate representative of 3 with similar results. Boxes delimit upper and lower quartiles, median values are indicated with a line, and single values are shown as dots. Bars indicate max/min values and **** statistical significance *P* value < 0.001 (one-way ANOVA using Dunnett’s multiple comparisons test). **h** Median longitudinal sections of WT roots with KOIN-GFP driven by *SCRp*. Cellular abbreviations: CEI cortex/endodermal initial, CEID CEI daughter, E endodermis, C cortex, Ep epidermis, X xylem axis, P pericycle. White arrowheads highlight KOIN-GFP localization to the inner lateral domain in the endodermis. Images displayed in this figure are representative of observations in experiments replicated at least 3 times with each replicate containing at least ≥15 plants/genotype. Scale bars: **a** 50 µm; **d**, **e**, **g** 20 µm.

Analysis of genes expressed downstream of SHR activation in the GT led to the identification of IRK and another LRR–RLK we named KOIN (encoded by At5g58300), as candidate proteins involved in the regulation of GT cell divisions^15,20–22^. *koin* alleles have a larger root meristem with increased stele area and more numerous cells in the longitudinal axis, as measured by cortex cell number and T-junction length, phenotypes consistent with excessive cell divisions (Fig. 1a–c; Supplementary Fig. 1). Although *koin* roots are larger than WT roots in the radial axis, we consistently observe the expected eight GT cells around the stele and no increase in overall root length (Supplementary Fig. 1f–h). Thus, IRK and KOIN both function to repress root cell proliferation but IRK restrict GT cell number in the radial axis, while KOIN operates in the longitudinal axis.

KOIN-GFP expressed from its endogenous promoter (*KOINp*) was detected in the root meristem in the endodermis, phloem pole pericycle (excluded from the xylem pole pericycle), and vascular tissues (Fig. 1d, e), but not detected in the outermost cell layers. Strikingly, in the endodermis and pericycle, KOIN-GFP accumulates asymmetrically at the inner PM domain, towards the stele, which is the opposite of IRK (Fig. 1d, e, h). This KOIN-GFP reporter rescues all the observed *koin* mutant root phenotypes (Fig. 1g; Supplementary Fig. 2). This indicates that KOIN is a polarized LRR–RLK whose activity interior to the cortex is sufficient to modulate cell division activity across the root cell layers. Interestingly, KOIN-GFP expressed from *SCRp* is polarized and largely rescues the *koin* root phenotypes (Fig. 1g; Supplementary Fig. 3). Therefore, KOIN activity in the endodermis is sufficient to restrict cell division across root cell types and control overall meristem size. In a similar situation *SCRp* driven IRK-GFP expression rescues *irk* root phenotypes^15^. Thus, polarized KOIN and IRK at the endodermis are functionally important for their activities downstream of SHR.

As both, KOIN and IRK, are polarized, we tested if similar orienting cues drive their localization in different cell types. IRK localizes to distinct polar domains in various root cell types and this polarity is oriented locally by cues from adjacent cell types, particularly in the GT (Supplementary Fig. 4a, d)^15^. In contrast, cell layer-specific expression of KOIN-GFP showed accumulation at the inner PM domain (stele-oriented) regardless of cell type (Fig. 1h, Supplementary Fig. 4a–g). This localization held true even in *scr-4*, which only has one GT cell layer with mixed endodermal and cortex identity (Supplementary Fig. 4d, h). These results indicate that, unlike IRK, KOIN polarity is inner/stele-oriented, regardless of cell type, suggesting orientation by a stele-derived cue. Therefore, different mechanisms determine the contrasting polarity of these receptors. Importantly, this contrasting polarity allows us to investigate the underlying mechanisms of LRR–RLK polar accumulation in the root GT and to connect polar receptor distribution to specific cell division control in the root.

### IRK and KOIN are differentially targeted to new PMs in dividing cells

**Fig. 2:**
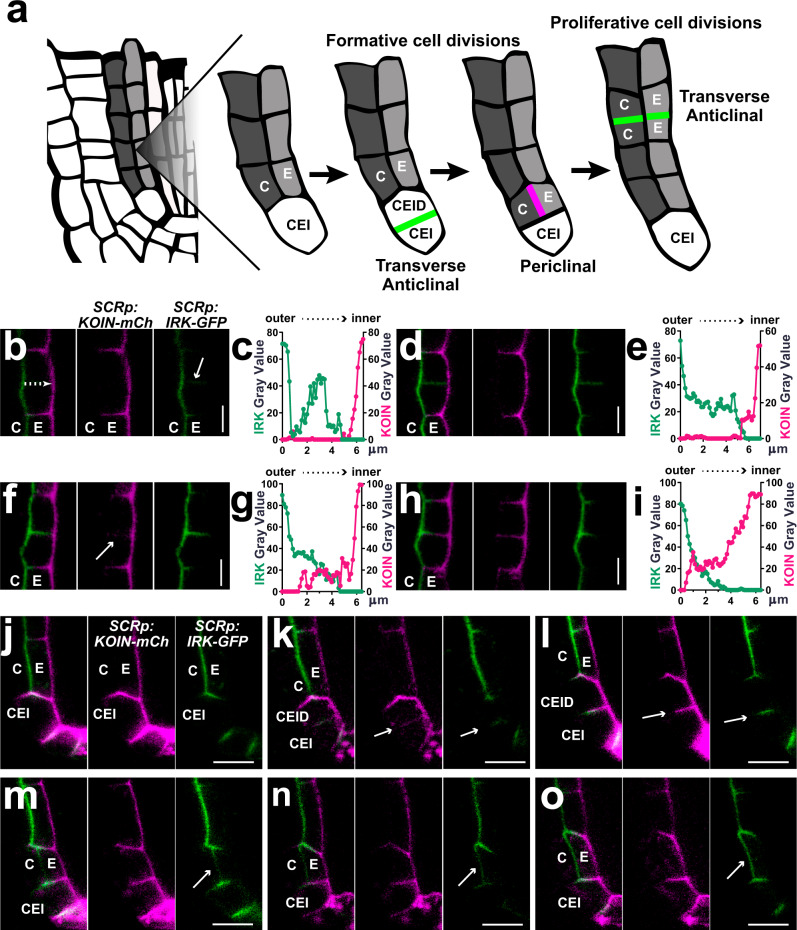
Dynamic polar distribution of KOIN and IRK upon GT cell divisions. **a** Schematics of a portion of a WT root tip highlighting formative and proliferative cell divisions during GT development. (Left to right) Formative transverse anticlinal division of the CEI (green) followed by periclinal formative division of the CEID (pink) to form endodermal and cortex cells. GT cells then proliferate through transverse anticlinal cell divisions (green). **b**, **d**, **f**, **h**, **j**–**o**) Adjacent panels show KOIN-mCherry (mCh, magenta) and IRK-GFP (green) driven by *SCRp* with fluorescence images merged and then each channel alone. **b**, **d**, **f**, **h** KOIN-mCh and IRK-GFP localization during proliferative transverse anticlinal endodermal cell divisions. **c**, **e**, **g**, **i** Graphs of fluorescence intensity gray values (arbitrary units) across the new PM represented in µm (dashed arrow as shown in (**b**): IRK-GFP (green lines) and KOIN-mCh (magenta lines). **j**–**o** KOIN-mCh and IRK-GFP localization in the CEI (**j**) before and (**k**–**l**) during formative transverse anticlinal cell division and in the CEID (**m**–**o**) during formative periclinal cell division. Note the CEI, cortex, and endodermal cells have elongated in (**o**). White arrows indicate fluorescence detected at new PMs. Scale bars: 10 µm. Images are representative of studies that were performed at least three times with at least 15 plants per condition per replicate.

During proliferative cell divisions, PIN2 is delivered to both sides of the new PMs by non-targeted secretion followed by post-cytokinetic (re-)establishment of shootward polarity in daughter cells^6,7^. To determine if this mechanism applies to KOIN and IRK, we examined their polarization during proliferative, transverse anticlinal divisions (Fig. 2a). As IRK and KOIN contrapolar endodermal accumulation are central for their root meristem functions, we examined *SCRp* driven IRK-GFP and KOIN-mCherry (mCh) in the same plants. IRK accumulation at newly formed PMs is detected first (Fig. 2a–e) with IRK-GFP fluorescence becoming progressively more intense towards the outer face and dimmer towards the inner face (Fig. 2a–e; Supplementary Fig. 5a–c). Then, KOIN becomes visible and is later detectable across new PMs with a stronger signal toward the inner endodermal face (Fig. 2f–i; Supplementary Fig. 5e, f). Therefore, during transverse anticlinal, proliferative endodermal cell divisions IRK and KOIN secretion is spatiotemporally targeted to new PMs, while lateral polarity remains undisturbed.

Unlike proliferative divisions, during formative asymmetric cell divisions, cellular components, such as organelles and proteins, are unequally distributed to the daughter cells^23^. Therefore, we examined IRK and KOIN distribution during and after CEI/CEID formative cell divisions (Fig. 2a), as this may contribute to the establishment of endodermal lateral polarity. In the CEI, KOIN is absent only from the outer PM domain, while IRK is restricted to the center of the shootward and rootward domains (Fig. 2j and Supplementary Fig. 5g–j). Upon formative, transverse anticlinal division of the CEI, both KOIN and IRK are detected at new PMs (Fig. 2a, k, l). However, IRK mainly accumulates at the center of these new membranes, while KOIN is highest at the inner side and declines towards the outer side (Supplementary Fig. 5k–n). Remarkably, upon the formative periclinal division of the CEID, only IRK is detected at the new PMs (Fig. 2m–o). This division plane coincides with the outer polar domain of newly formed endodermal cells and KOIN is not detectable at this domain. Subsequently, the inner/outer PM localization of KOIN and IRK is maintained as endodermal cells undergo further proliferative divisions. Therefore, the inner/outer polarity of the endodermal daughter cell is directly influenced by CEID-specific sorting of IRK and exclusion of KOIN from the new PMs as division occurs.

To gain insight into the relationship between periclinal divisions of the CEID and IRK lateral polarization we used, as a tool, *pub4-1*, a mutant with a greater number of CEI/CEID^24^. We delayed the formative division of the CEID by treating *pub4-1* with the chemical Brefeldin A (BFA)^25^. After 5 h, and more evidently after 24 h treatments, undivided CEIDs were interspersed with newly formed endodermal cells (Supplementary Fig. 5o, p). Rootward/shootward IRK-GFP localization in the CEID was maintained until the CEID divided, then IRK polarization to the outer PM domain in the endodermis was established and remained unaltered after further divisions (Supplementary Fig. 5o). These results further demonstrate a link between the formative, periclinal division of the CEID and the establishment of IRK lateral polarity.

### Distinct secretion pathways direct IRK and KOIN polarization

Determining KOIN-GFP and IRK-GFP dynamics may help us understand the mechanisms underlying the regulation of polarity and its relationship to protein function. Analysis of fluorescence recovery after photobleaching (FRAP) can reveal differential dynamics of polarized proteins, which may arise from protein secretion, recycling, lateral diffusion, and/or local interactions^8,26^. After photobleaching, faint signal recovery (less than 15%) was observed for both KOIN-GFP and IRK-GFP, suggesting that these PM proteins have little to no lateral diffusion and that the initial pool of proteins is replaced at a slow rate (Supplementary Fig. 6a-j). Many polarized PM proteins have a low recovery rate^3,7^ and appear relatively stationary, yet IRK and KOIN recovery appear to be slower even among them.

**Fig. 3:**
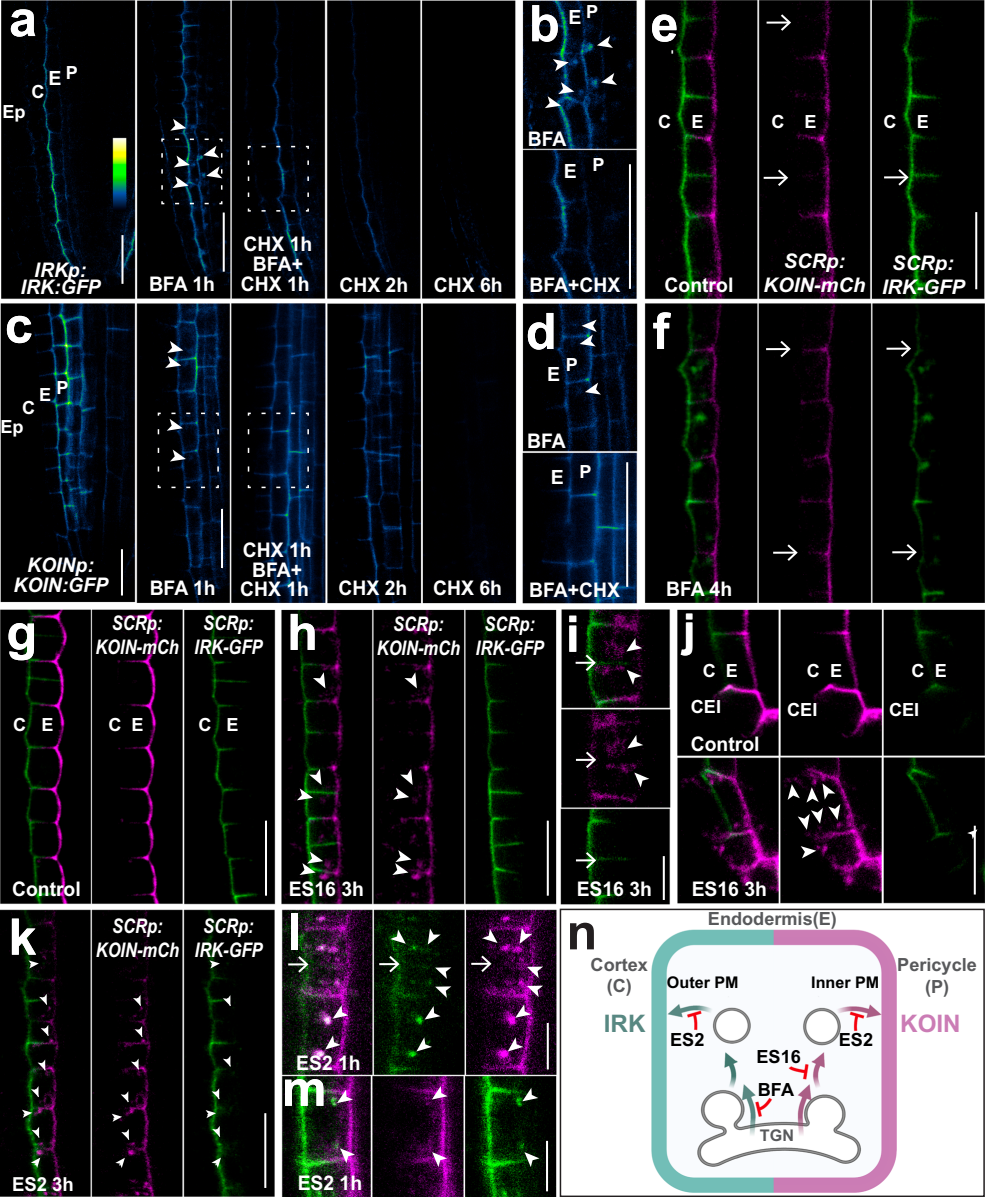
Chemical treatments assessing endomembrane trafficking contributions to IRK and KOIN polar distribution. **a**, **c** Confocal images of WT roots expressing *IRKp:IRK-GFP* and *KOINp:KOIN-GFP* with GFP shown in fluorescent intensity color scale. Roots treated with DMSO (control), 50 µM BFA for 1 h (squares indicate magnified images in (**b**) and (**d**)), 50 µM CHX for 1 h followed by a co-treatment 50 µM BFA and 50 µM CHX for an additional 1 h, and finally, treatment with 50 µM CHX for 2 and 6 h. White arrowheads indicate BFA bodies. **e**, **f** Confocal images (merged) of roots expressing IRK-GFP (green) and KOIN-mCh (magenta) from *SCRp* treated with (**e**) DMSO (control) and (**f**) 50 µM BFA for 4 h. White arrows indicate the differential distribution of IRK and KOIN at new PMs. **g**–**i**, **k**–**m** Endodermal cells and (**j**) CEI expressing *SCRp* driven IRK-GFP (green) and KOIN-mCh (magenta) with merged images followed by single channels. **g** Control (DMSO) conditions, **h**, **i** treated with 15 µM ES16 for 3 h. Upon ES16 treatment KOIN-mCh agglomerations (arrowheads) are observed adjacent to the forming cell plate where IRK-GFP is already accumulating (white arrows). **j** CEI in control conditions or after 3 h treatment with 15 µM ES16. White arrowheads indicate KOIN agglomerations in (**h**, **i**) endodermal cells near the transverse anticlinal division plane and in (**j**) the CEI and after the periclinal division of the CEID. **k**–**m** Endodermal cells of roots treated with 40 µM ES2 for 3 h. Arrowheads indicate ES2-induced agglomerations of IRK-GFP and KOIN-mCh. **l** The arrows indicate forming PMs and the arrowheads show the ES2 agglomerations. **n** Schematic of an endodermal cell with the cortex (left) and pericycle (right) summarizing chemical treatment results (created at BioRender.com). IRK (green) and KOIN (purple) trafficking to the outer and inner domains of the PM, respectively. IRK and KOIN are secreted from different TGN domains. IRK is secreted through a BFA- and ES2-sensitive pathway, while KOIN is secreted through an ES16- and ES2-sensitive pathway. Scale bars: 15 µm (**a**–**h**, **j**, **k**); 5 µm (**i**, **l**, **m**). Images are representative of observations in experiments replicated at least 3 times with each containing at least 15 plants/conditions.

To further characterize IRK and KOIN protein dynamics we used a set of chemicals that interfere with protein trafficking at distinct points in the secretory and endocytic pathways (summarized in Supplementary Table 1). BFA partially interferes with protein secretion by targeting a subset of guanidine exchange factors (GEFs) acting on ARF-GTPases (ARF-GEFs) resulting in intracellular agglomerations called BFA bodies that co-localize with trans-Golgi network (TGN) markers^27–30^. One-hour BFA treatment (50 µM) led to intracellular IRK-GFP accumulation in BFA bodies but did not affect its polar distribution (Fig. 3a, b, e, f; Supplementary Fig. 7a). To determine whether IRK-GFP accumulation at BFA bodies corresponds to proteins trafficked after de novo synthesis and/or proteins cycling back to the PM after endocytosis (recycling), we pre-treated with the protein synthesis inhibitor cycloheximide (CHX) followed by co-treatment with BFA. IRK-GFP was depleted from BFA bodies indicating that the GFP signal corresponds to interference with trafficking of newly synthesized protein (Fig. 3a, b). Accordingly, extended CHX treatments nearly depleted IRK-GFP signal from the PM indicating that newly synthesized protein is the main contributor to IRK polar accumulation and that the pool of PM protein present prior to the treatment was degraded (Fig. 3a). In contrast, a substantial fraction of KOIN-GFP traffic is not affected by BFA, as only faint agglomerations were visible in a few pericycle cells, and which disappeared upon pre-treatment with CHX (Fig. 3c, d). CHX treatments decreased KOIN-GFP accumulation at the PM and the signal was nearly undetectable after 6 h pointing to degradation of the existing PM protein pool. Thus, CHX treatments indicate that the polarization of both proteins is predominantly dependent on the secretion of the newly synthesized protein. Additionally, accumulation of IRK, but not KOIN, at newly formed PMs after proliferative endodermal cell divisions was also sensitive to BFA treatment (Fig. 3e, f, n). These results show that a BFA-insensitive secretion pathway is a principal contributor to KOIN polarity.

As IRK and KOIN polar endodermal accumulation is sufficient for function, we continued to analyze mechanisms of polarization specifically in this cell type using a complementary set of compounds previously shown to affect protein polarization. Disruption of the microtubule or actin cytoskeleton did not alter the endodermal polar distribution of BOR1 and NIP5;1^10^ or IRK and KOIN (Supplementary Fig. 7b, c). Additionally, no change was observed upon treatment with Wortmannin (Wm), an inhibitor of phosphoinositol synthesis, which affects lateral polarization of NIP5;1 in the endodermis^10^. However, extended Wm treatment decreased IRK and KOIN accumulation at the PM (Supplementary Fig. 7f, g), indicating that phosphoinositol synthesis contributes to their stability or delivery.

We next treated the roots with Endosidin16 (ES16), which alters shootward PIN2, but not rootward PIN1 secretion by targeting the RAB GTPase A2A (RABA2A)^5^. Interestingly, ES16 affected secretion of KOIN, but not IRK, to newly formed PMs during proliferative and formative cell divisions (Fig. 3g–j). Thus, an ES16 sensitive mechanism is involved in the secretion of KOIN, but not of IRK, which could explain temporal differences in their accumulation at newly formed PMs (Fig. 3n). We also treated the roots with Endosidin2 (ES2), which produces intracellular PIN2 agglomerations by targeting EXO70A1 and interfering with polar secretion from the TGN to the PM^31^. ES2 treatment caused intracellular IRK and KOIN agglomerations, particularly near the division plane (Fig. 3k–m), suggesting targeting of both proteins to new PMs requires EXO70A1. Furthermore, extended ES2 treatment significantly reduced the overall accumulation of IRK and KOIN at the PM (Supplementary Fig. 7d, e). This depletion indicates that pools of PM-localized IRK and KOIN, present prior to ES2 treatments, were degraded. Consistent with this, ES2 was shown to enhance PIN2 endocytic traffic to the lytic vacuole^32^. IRK and KOIN trafficking to the lytic vacuole is also evidenced by their retention in doughnut-shaped agglomerations induced by Wm interference with degradative trafficking (Supplementary Fig. 7h). However, the Wm-induced IRK and KOIN accumulations did not always co-localize, suggesting differential timing of their transport to the vacuole.

Altogether, our results show that IRK and KOIN polarization is driven by polar secretion of newly synthesized protein through distinct pathways that confer specificity of cargo delivery to the cell division plane and/or the lateral membranes (Fig. 3n).

### KOIN protein sorting and function require its kinase domain

**Fig. 4:**
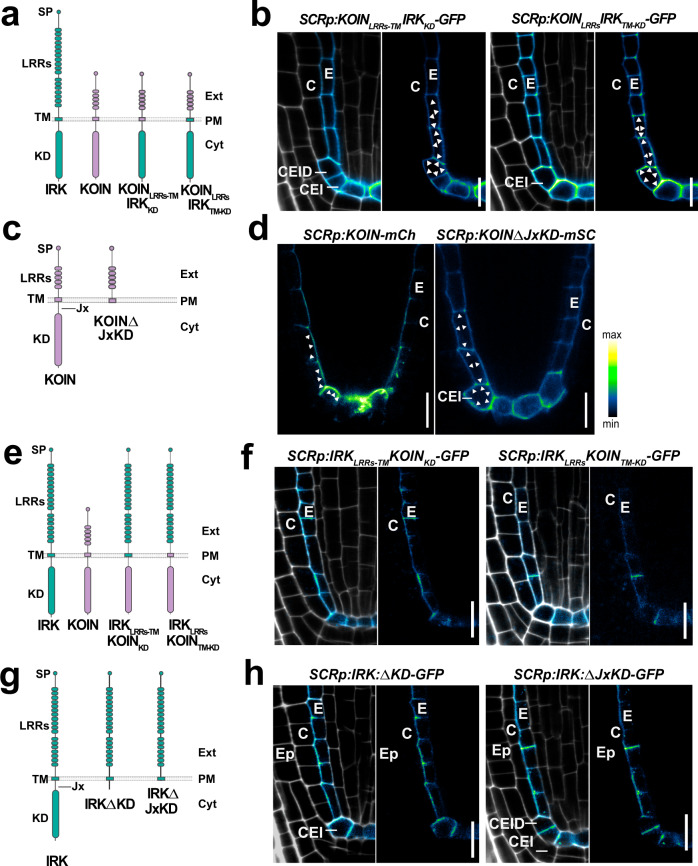
KOIN and IRK sorting determinants reside in different portions of the protein. **a** Diagrams of IRK (green) and KOIN (purple) proteins with their extracellular signal peptide (SP) and LRR domains, transmembrane (TM) domain, and kinase domain (KD), and the KOIN-IRK protein chimeras. **b** Confocal micrographs showing WT root tips expressing *SCRp* driven KOIN-IRK chimeras with adjacent panels showing GFP + PI merged or GFP alone (GFP intensity color scale, PI gray). Arrowheads indicate the non-polar distribution of this chimera. **c** Diagrams of KOIN full-length and C-terminal truncation missing the juxtamembrane (Jx) domain and Jx and KD (JxKD). **d** Confocal images WT root tips expressing *SCRp*-driven KOIN-mCh (left) and KOINΔJxKD-mSCarlet (mSC, right) with FP shown in intensity color scale. Arrowheads indicate protein distribution. **e** Diagrams of IRK (green) and KOIN (purple) protein domains and the IRK-KOIN chimeras. **f** Confocal images of WT root tips expressing *SCRp* driven IRK-KOIN chimeras and stained with PI (gray) (merged, left) and GFP fluorescence alone (intensity color scale, right). **g** Diagrams of IRK full-length and C-terminal truncations missing the KD or the Jx and KD (JxKD). **h** Confocal images of WT root tips expressing *SCRp* driven IRK truncations with adjacent panels showing GFP + PI merged (left) or GFP alone (right). The images are representative of the observations from experiments performed at least three times with at least 15 plants per genotype and at least 3 independent transgenic lines were analyzed. Scale bars: 10 µm. Abbreviations: (Ep) epidermis; **c** cortex; **e** endodermis, (CEI) cortex/endodermal initial, and (CEID) CEI daughter.

Studies in mammalian and yeast systems indicate that several transmembrane proteins contain cytosolic sorting motifs recognized by adapter proteins that facilitate their delivery to specific destinations^33–35^. However, studies on polar sorting motifs in plants are less advanced^13,14,36^. We reasoned that polar sorting of IRK and KOIN may be determined by distinct protein-specific domains and investigated this using a molecular domain swapping approach. LRR–RLKs are composed of an extracellular domain containing multiple LRRs, a transmembrane domain (TM), and a cytoplasmic kinase domain (KD) (Fig. 4a). First, we tested if KOIN localization was disrupted by swapping the KOIN TM and kinase domains with those from IRK to generate KOIN_LRRs_IRK_TM-KD_-GFP and KOIN_LRRs-TM_IRK_KD_-GFP chimeras (Fig. 4b). When expressed under the control of the *SCRp* in WT both chimeras showed nonpolar localization in endodermal, CEI/CEID, and QC cells (Fig. 4c). Thus, the KOIN extracellular domain is not sufficient to direct its polarization. Consistent with this, a KOIN truncation lacking the LRR domains (ΔLRRs) is largely retained in endomembrane compartments, but the portion that reaches the PM accumulates to the inner polar domain (Supplementary Fig. 8a–c).

To test if the KOIN cytoplasmic domains were responsible for its polar localization, we truncated KOIN removing the juxtamembrane (Jx) and KD (KOIN∆JxKD), and found that it had nonpolar accumulation at the PM (Fig. 4c, d). Therefore, KOIN sorting determinants are contained in the cytoplasmic domains and, remarkably, in the absence of these domains, KOIN protein secretion lacks specificity and is misdirected with nonpolar accumulation in endodermal, CEI/CEID, and QC cells. Given its nonpolar accumulation, we also investigated whether KOIN∆JxKD could rescue *koin* root phenotypes. However, endodermal-expressed KOINΔKD-mSC in *koin* remains nonpolar and, in contrast to full-length KOIN, fails to rescue *koin* root phenotypes (Supplementary Figs. 4 and 9). These results indicate that the KOIN KD is required for it to be actively recognized and sorted into a targeted polar secretion pathway and to function to repress root cell divisions.

### IRK polar sorting determinants reside in the extracellular domain

As the KOIN intracellular domain is sufficient for its polarity, we determined if this precedent could be extended to IRK. We first fused the IRK extracellular and TM domains to the KOIN KD generating the IRK_LRRs-TM_KOIN_KD_-GFP chimera (Fig. 4e). Upon expression by *SCRp* in WT, this chimera accumulates like IRK to the outer PM domain, and a similar localization was observed for the IRK_LRRs_KOIN_TM-KD_-GFP chimera (Fig. 4f).

IRK and KOIN are classified as atypical or pseudo-kinases because they contain non-conserved residues at key positions in their KDs (Supplementary Fig. 10a), and they are reported to lack kinase activity^37–40^. We next determined how polar sorting was affected by the fusion of the active KD of the LRR-RLK ERECTA^41^ to the IRK extracellular and TM domains (Supplementary Fig. 10b, c). Again, IRK_LRRs-TM_ERECTA_KD_-GFP chimera exhibited outer lateral polar distribution in the endodermis. Thus, in the endodermis, the IRK extracellular domain is the dominant determinant directing the protein to the outer polar domain, while the TM or KD of IRK are exchangeable, regardless of KD activity status.

To further dissect the contribution of the IRK extracellular domain to polarity, we performed a series of deletions of LRR domains. However, all IRKΔLRR truncations were retained at the endoplasmic reticulum (ER, Supplementary Fig. 8d–h). This indicates that the exit of IRK from the ER requires precise quality control of the structure of its extracellular domain. To continue assessing the extracellular domain’s importance in determining IRK polarity, we created truncated versions lacking the KD (IRKΔKD-GFP) or the entire cytoplasmic domain (Jx and KD; IRKΔJxKD-GFP) (Fig. 4g, h). These IRK truncations retained their polar accumulation in the CEI/CEID and endodermis and when expressed under the control of *CO2p* or *IRKp* were distributed similarly to full-length IRK (Supplementary Fig. 10d–g). Therefore, truncated IRKΔKD and IRKΔJxKD are recognized and correctly sorted in different cell types. Thus, contrary to our knowledge in other systems^24,25,35^, the IRK extracellular, not the cytoplasmic domain, carries the sorting determinants for its polarity. Collectively, our evidence indicates that in plants at least two different sorting mechanisms directing LRR-RLK polarization exist, one recognizing extracellular motifs and another recognizing cytoplasmic motif.

### IRKΔKD is polarized and partially rescues phenotypes in *irk-4*

**Fig. 5:**
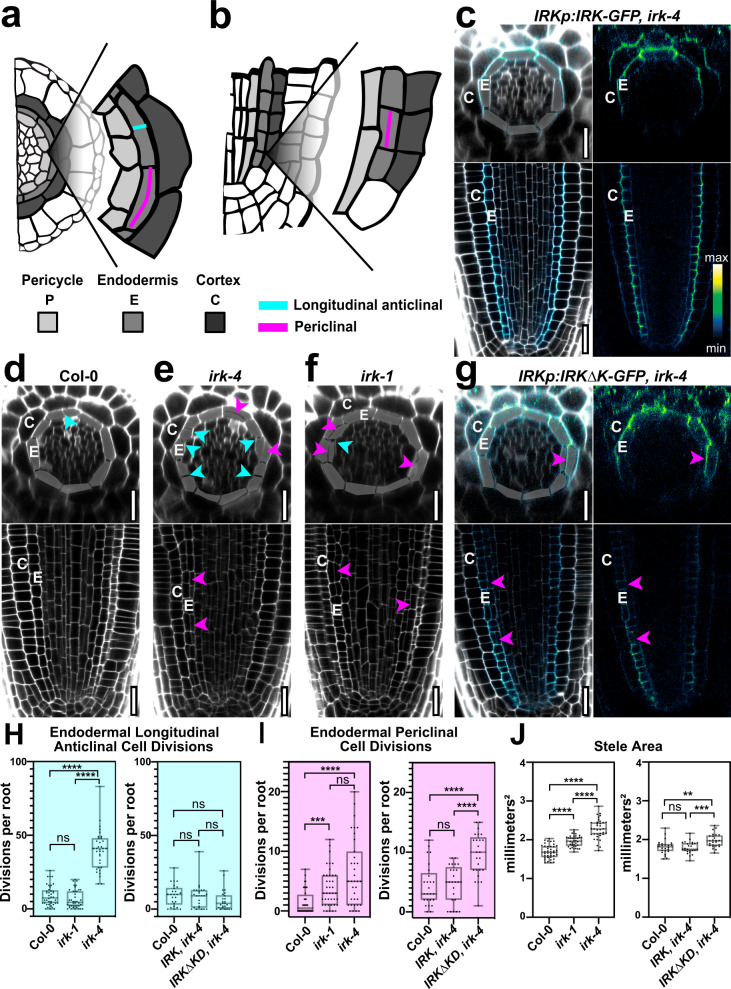
IRK*∆*KD is polarized in and partially rescues the cell division phenotypes in *irk-4*. **a**, **b** Schematics of portions of the Arabidopsis root tip in **a** transverse and **b** median longitudinal sections. **c**–**g** Confocal images of transverse (upper, with endodermal cells highlighted) and median longitudinal (lower) optical sections from root tips stained with PI (gray) and **c**, **g** GFP with intensity color scale and PI (merged) and GFP fluorescence alone. **c** *irk-4* root expressing *IRKp:IRK-GFP*. **d**–**f** Roots of WT, *irk-4*, and *irk-1*. **g** *irk-4* roots expressing *IRKp:IRK∆KD-GFP*. Endodermal longitudinal anticlinal (cyan) and periclinal (magenta) cell divisions are indicated. Quantification of **h** longitudinal anticlinal and **i** periclinal endodermal cell divisions and (**j**) stele area of WT, *irk-4* and *irk-1* roots and *irk-4* roots expressing IRK or IRK***∆***KD fusions under *IRKp* (*n* = 20 roots per genotype). Graphs **h**–**j** show representative results of experiments performed in ≥3 independent replicates with similar significant results, boxes delimit upper and lower quartiles, median values are indicated with a line, and single values are shown as dots. Bars indicate max/min values and 1–4 stars statistical significance *P* value < 0.05 (Brown–Forsythe one-way ANOVA test). Scale bars: 20 µm.

In mammalian cells, homodimerization and clustering of certain single-pass transmembrane receptors are linked to the establishment of lateral polarity^42,43^. In plants, LRR-RLKs are putative ligand-binding receptors and act as co-receptors by homodimerizing or interacting with other proteins through their extracellular, TM, or cytoplasmic domains^44,45^. As IRKΔKD-GFP shows the polar distribution in WT, we wanted to eliminate the possibility that homodimerization or clustering with endogenous IRK was the driver of IRKΔKD-GFP polarization. In the absence of endogenous IRK (in *irk-4*) truncated IRKΔKD retained its polar localization (Fig. 5g) and, remarkably, endodermal or endogenous expression of IRK∆KD-GFP rescued the *irk-4* endodermal LAD phenotype. The IRK∆KD-GFP roots still showed additional periclinal cell divisions and an enlarged stele (Fig. 5c, g–j: Supplementary Fig. 11), which is similar to the phenotypes of *irk-1* (Fig. 5f, h–j), a weaker, insertional allele with a premature stop codon that, if translated, would be comparable to IRK∆KD^15^. Furthermore, only expression of *IRK-GFP* fully rescued the increased endodermal periclinal cell divisions, increased stele area, and LADs observed in *irk-4*^15^ (Fig. 5a, b, d, e, h–j). These results indicate that the IRK extracellular domain directs polarization at the PM and in the absence of the KD retains partial function in the repression of longitudinal anticlinal divisions of endodermal cells.

### The KOIN/IRK chimera enhances *irk* root patterning defects

**Fig. 6:**
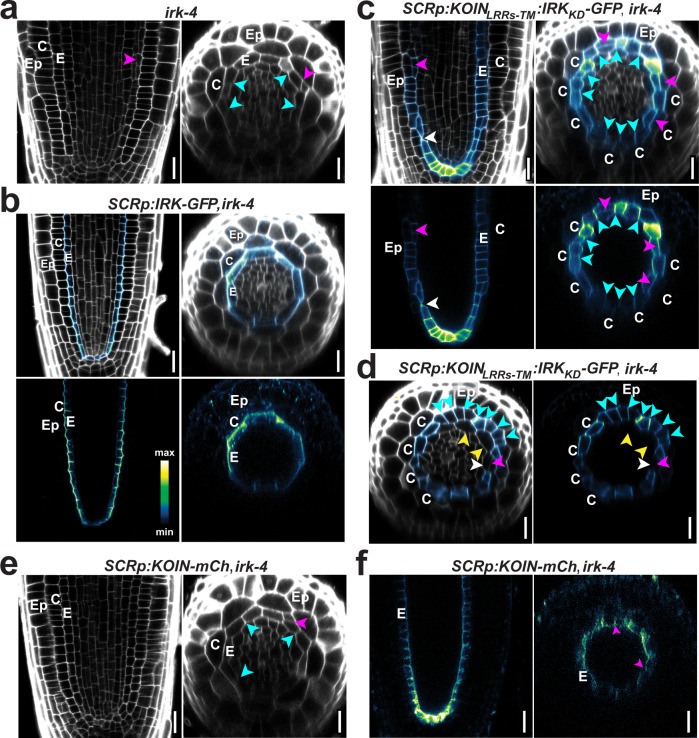
Endodermal expression of KOIN-IRK chimera results in additive cell division phenotypes in *irk-4*. **a**–**f** Confocal images of median longitudinal and/or transverse sections of roots stained with PI (gray) and/or fluorescence (intensity color scale). **a** *irk-4* roots showing endodermal longitudinal anticlinal (cyan arrowheads) and periclinal (magenta arrowheads) cell divisions. **b** *irk-4* roots expressing *SCRp:IRK:GFP* (GFP + PI merged) and GFP alone. **c**, **d** *irk-4* roots expressing *SCRp:KOIN*_*LRRs*_*IRK*_*TM-KD*_*-GFP* at **c** 7 and **d** 4 d.p.s. showing GT disorganization. *SCRp* driven GFP signal is detected in cells that positionally correspond to the cortex as they are immediately adjacent to the epidermis but have undergone many longitudinal anticlinal cell divisions. White arrowheads indicate an apparent discontinuity in endodermal cell files/rings. **d** A root with cells immediately interior to the epidermis that express the *SCRp* driven chimera with cells interior to those that morphologically resemble cortex (yellow arrowheads). **e**, **f** *irk-4* roots expressing *SCRp:KOIN-mCh* **g** stained with PI (gray) and showing **h** KOIN-mCh localization (mCh intensity false-colored). Images correspond to representative observations of experiments replicated at least 3 times using ≥15 plants/genotype. Scale bar: 20 µm.

Our results indicate that the IRK cytoplasmic domain is dispensable for polarization, but necessary to repress the endodermal periclinal divisions and stele enlargement in *irk-4*. To further assess the functional relevance of IRK-KD in the *irk-4* background, we analyzed *SCRp*-driven endodermal expression of the nonpolar KOIN_LRRsTM_IRK_KD_-GFP chimera (Fig. 6; Supplementary Fig. 12f). The dysregulated endodermal cell divisions of *irk-4* were not rescued, and additional cellular phenotypes were observed. In the longitudinal axis, cell files with *SCRp* activity appear discontinuous (Fig. 6c, d). In the radial axis, cells with *SCRp* activity are adjacent to cells that appear epidermal by position and morphology. Coincidentally, many of those *SCRp*-expressing cells have also undergone repeated LADs (Fig. 6c, d; Supplementary Fig. 12f). It is possible that excessive endodermal LADs lead to the displacement of the cortex cells resulting in a gap, where endodermal and epidermal cells are immediately adjacent. However, many of these *SCRp-*expressing cells, based on their position and (pre-division) morphology, appear to have once been cortex cells, suggesting the fate of those cells has been altered. In either case, these results indicate disorganization of root GT, including boundary disruption between cell types. Furthermore, cells with cortex morphology unexpectedly occur interior to *SCRp*-expressing cells (yellow arrowheads, Fig. 6d). These observations are consistent with general disorganization of longitudinal and radial tissue patterning. Notably, expression of the KOIN_LRRsTM_IRK_KD_-GFP chimera in WT or *koin-1* does not generate additional abnormal phenotypes indicating that this enhanced disorganization occurs specifically in *irk-4* (Fig. 4f and Supplementary Fig. 12d, f).

We were able to exclude that the non-polar KOIN extracellular domain was causal for the phenotypic enhancement of *irk-4* (Supplementary Fig. 12a, f). Additionally, expression of full-length KOIN from *SCRp* in *irk-4* did not result in enhanced phenotypes (Fig. 6e, f), nor did IRK expression in *koin-1* (Supplementary Fig. 12c, e, f). Hence, we reasoned that these remarkably enhanced phenotypes can be fully attributed to the expression of KOIN_LRRsTM_IRK_KD_ in *irk-4*. The chimeric nature of the protein suggests that in *irk-4* the non-polarly localized KOIN LRRs perceive signals that are wrongly transduced by a non-polar IRK-KD, leading to disorganized cell layers on either side of the endodermis, which disrupts root radial patterning.

The defects in cell division and tissue patterning generated by the chimera together with the misregulated cell division phenotypes of *koin* and *irk* single mutants supports a hypothesis where bi-directional cues converge on the endodermis and are perceived by polarized receptors that modulate specific cell divisions and participate in coordinating cell proliferation and patterning across the entire root meristem.

## Discussion

The establishment and maintenance of polar protein localization at the PM during dynamic cellular events, such as cell division, remain a matter of study. Upon proliferative epidermal cell divisions PIN1, PIN2, and BOR1 are trafficked to the new PMs by non-polar secretion. After cytokinesis, the processes of endocytosis, recycling or degradation, and secretion of newly synthesized proteins, finally, establish shootward/rootward polarity of PIN1/PIN2 and lateral polarity of BOR1^4,6,7,46^. In contrast to these, we find that during proliferative divisions IRK and KOIN have different spatiotemporal accumulation at new PMs indicating distinct targeted secretion mechanisms. A battery of regulatory proteins act at different stages of cell plate formation and are likely involved in spatiotemporal differences in the delivery of specific cargos^47–49^. Additionally, post-cytokinetic polarization of IRK and KOIN depends only on the targeted secretion of de novo protein with their polar delivery occurring via differentially regulated secretion pathways.

During the periclinal formative division of the CEID, IRK secretion is targeted to new PMs while KOIN is not, thus determining the asymmetric accumulation of these two proteins in the daughter endodermal cell. Our observations are consistent with an active role for the mother cell (the CEID here) in the specific sorting and secretion of proteins to new PMs. The resulting polar distribution of these LRR–RLKs in endodermal daughter cells is maintained as they proliferate in the root meristem.

Polar protein secretion is coordinated by small GTPases, the EXOCYST vesicle tethering complex, and pairs of cognate SNAREs on opposing membranes^26^. We find that IRK secretion proceeds through a BFA-sensitive and ES16-insensitive pathway, contrary to KOIN, which is secreted by a BFA-insensitive and ES16-sensitive pathway (Fig. 3n). BFA targets certain ARF-GEFs impacting ARF small GTPase activity and vesicle formation in the Golgi and certain TGN subdomains, which affects the secretion of numerous cargos during and after cell division^50–54^. ES16 targets the small GTPase RABA2A affecting secretion from certain TGN subdomains during interphase and cytokinesis^5,55^. Therefore, KOIN and IRK secretion appear to be sub-compartmentalized into different TGN subdomains. Accordingly, the existence of molecularly, functionally, and spatially distinct TGN subdomains have been experimentally demonstrated^56^. Compartmentalized secretion from the TGN is reported for a handful of polarized proteins^5,55,57^ and contributes to explaining the differential accumulation of IRK and KOIN at the PM during and after proliferative cell divisions.

Trafficking and targeting of TGN-derived vesicles to the appropriate PM domain involve the multiprotein EXOCYST complex^58,59^. Secretion of IRK and KOIN is sensitive to ES2 indicating the involvement of the EXOCYST subunit EXO70A1. This subunit regulates polar exocytosis of a subset of PM proteins indicating selectivity^31,32^. Interestingly, extended treatments with ES2 and Wm, a phosphatidylinositol 3-(PtdIns3K) and 4-(PtdIns4K) kinase inhibitor, decreased IRK and KOIN accumulation at the PM. Wm-induced interference with PtdIns synthesis depletes EXO70A1 from the PM^60,61^. EXO70A1 binds to phosphatidylinositol 4-phosphate (PtdIns4P), which is constitutively present at the PM and expanding cell plates^62,63^. Therefore, a Wm-induced decrease in PtdIns4P may affect EXO70A1 positioning at the PM and, consequently, the targeted secretion of IRK and KOIN. Thus, we propose that IRK and KOIN are secreted through distinct pathways that converge at EXOCYST-mediated targeting.

Secretory proteins contain sorting motifs deciphered by the transport machinery to route them into specific secretion pathways^64,65^. In mammalian systems, sorting signals determining polar secretion are found within the cytoplasmic and TM domains of different single-pass TM proteins^66,67^. Likewise, KOIN sorting determinants are present in its cytoplasmic domain. However, none of the canonical cytoplasmic sorting motifs described in plants^14,65^ or other systems^68^ are present within the KOIN cytoplasmic domain indicating that specific, novel motifs direct its polar accumulation. Additionally, although KOIN is described as an inactive kinase^69^, its cytoplasmic domain is essential for function.

IRK sorting determinants, unexpectedly, reside in its extracellular domain. Therefore, a luminal recognition system must exist in the plant endomembrane system to properly direct the polar accumulation of IRK∆KD. IRK has 18 LRRs in the extracellular domain that likely participates in protein-protein interactions, therefore luminal associations may occur in the secretory pathway and assist delivery of IRK∆KD to the PM. Such is the case for FERONIA, a transmembrane RLK, that interacts with two glycosylphosphatydilinositol-anchored proteins (GPI-AP) at the ER for efficient secretion of the complex to the PM^70^. FERONIA is retained in the ER in the absence of GPI-AP or upon deletion of specific extracellular domains required for GPI-AP interaction, suggesting they act as chaperones facilitating ER exit. Deletions in the IRK extracellular domain also result in ER retention. Further research is necessary to determine if a similar chaperone-driven mechanism facilitates IRK exit from the ER.

The chimeras IRK_LRRs-TM_KOIN_KD_-GFP and IRK_LRRs_KOIN_TM-KD_-GFP showed IRK-like polarization, demonstrating the hierarchical importance of the sorting signals in the IRK extracellular domain over those found in the KOIN KD. These results reveal fascinating paths for further investigation into the precise mechanisms that recognize these sorting determinants.

Once polarized at the PM, the interaction of IRK∆KD with other proteins or extracellular components likely explains its ability to rescue the endodermal LADs, but not the periclinal cell divisions or enlarged stele phenotypes of *irk-4*. Strikingly, the *irk-1* allele, which is predicted to express an IRK∆KD-like protein, exhibits similar phenotypes and, like *irk-4*, shows altered *CYCD6;1p* activity. This indicates that, although IRK is described as an inactive kinase^69^, its KD is functionally important; consequently, only full-length IRK completely rescues *irk-4* root phenotypes. These results indicate partitioning of IRK function where putative interactions through the extracellular domain repress endodermal LADs, while those of the IRK cytoplasmic domain regulates periclinal endodermal divisions and stele enlargement.

Endodermal expression of the non-polarly localized KOIN_LRRs-TM_IRK_KD_-GFP chimera in *irk-4* results in cellular disorganization within and outside the endodermis, suggesting a non-cell-autonomous influence on cell division and perhaps cell identity. This chimera may inappropriately perceive signals due to its nonpolar localization and/or elicit an incorrect downstream response impacting root radial patterning. However, further research is necessary to fully establish this hypothesis. As IRK and KOIN are implicated in cell division control at the endodermis, it is tempting to propose that downstream of the SHR transcriptional network these two LRR–RLKs, polarized at opposites sides of the endodermal PM, interpret bi-directional information that modulates root cell proliferation in the radial and longitudinal axes. Our observations are consistent with others that indicate the endodermis non-cell autonomously regulates developmental events in the root meristem^71,72^. Identification of the extra- and intracellular interactors of IRK and KOIN will be critical to further untangle the precise developmental roles of these polarized LRR–RLKs.

## Methods

### Plant material and growth conditions

*Arabidopsis thaliana* ecotype Col-0 was used as wild type (WT). The *koin-1* (WiscDsLox_439H07) and *koin-2* (Gabi-Kat822B12) alleles were obtained from the ABRC (Arabidopsis Resource Center). The genotypes *IRKp:IRK:GFP*, *SCRp:IRK-GFP, CO2p:IRK-GFP, pub4-1 SCRp:IRK:GFP, irk-4, irk-1, SCRp:IRK:GFP irk-4, IRKp:IRK:GFP irk-4, pSCR:erGFP, scr-4* were previously described^15^. Unless otherwise specified, seeds were surface sterilized by chlorine gas, sown on solid Arabidopsis medium 1X Murashige and Skoog basal salts (Caisson labs), 0.5 g/L MES, 1% sucrose, and 1% agar (Difco), at pH 5.7; then stratified at 4 °C for at least 2 days prior to transfer to a 16/8 h illumination regime in a Percival growth chamber at a continuous temperature of 22 °C. Seedlings were grown on vertically oriented plates sealed with parafilm or micropore tape for 4–7 days prior to analysis.

### Plasmid construction

Expression vectors (Supplementary Table 2) were constructed utilizing Invitrogen Multisite Gateway^®^ technology (Carlsbad, USA). Briefly pENTR vectors containing the various promoters, coding regions, and fluorescent tags were recombined into the destination vectors to generate plant expression vectors for transformation.

mSCARLETi (in pUC57) was received from Dr. David Nelson (University of California, Riverside). The *mSCARLET* coding sequence (754 bp) was amplified with attB2/attB3 recombination sites added in the primers (Supplementary Table 3); then, recombined via BP clonase (Invitrogen, Cat #11789-013) into the pDONR^TM^ P2r-P3 vector.

KOIN is encoded by At5g58300 and all components were cloned by PCR from WT genomic DNA and verified by sequencing. The *KOIN* promoter (1709 bp) fragment containing the entire intergenic region between the stop codon of At5g58290 and the *KOIN* start codon was amplified (Supplementary Table 3) and recombined into the pENTR^™^ DIRECTIONAL TOPO^®^ (pENTR-D-TOPO) vector. The 2048 bp *KOIN* genomic region, including intron 2 (86 bp) and the coding region, was amplified (Supplementary Table 3) and recombined into the pENTR-D-TOPO vector.

To generate IRK extracellular domain deletions the pENTR-D-TOPO vector containing the *IRK* genomic sequence was digested with restriction enzymes to remove specific regions. Digested vectors were purified, re-ligated using Promega T4-DNA ligase, and the reading frame was verified by DNA sequencing. Specifically, IRKΔLRR1 was generated by digestion with XbaI, IRKΔLRR17,18 by digestion with SalI and SmaI, and IRKΔLRR1-18 by digestion with BglII and SmaI. In the case of IRKΔLRR17,18 and IRKΔLRR1-IRKΔ18, DNA polymerase I, large (Klenow) fragment (New England Biolabs) was used to fill in the overhangs prior to ligation. The KOIN extracellular domain deletion was similarly generated by digestion and ligation. To generate the SCRp:KOINΔLRR:GFP expression vector, the expression vector containing full-length KOIN-GFP driven by *SCRp* (*SCRp:KOIN:GFP)* was digested with EcoRI and AccIII (BspEI), purified, then overhangs were filled in with Klenow, and ligated together. Deletions of IRK cytoplasmic domains (IRKΔKD and IRKΔJxKD) were cloned by PCR from WT genomic DNA using primers designed to amplify from the IRK start codon up to the beginning of the KD (∆KD, 2115 bp, 674 aa) or just through the transmembrane domain (ΔJxKD, 2007 bp, 638 aa) and the fragments were recombined into the pENTR-D-TOPO vector. The cytoplasmic KOIN deletion KOINΔJxKD was similarly generated by amplifying the region from its start codon through the transmembrane domain (∆JxKD, 942 bp, 314 aa).

To create the different chimeric proteins, DNA fragments of interest were cloned by PCR from cDNA clones (IRK: N3G56370ZEF, KOIN: N5G58300ZEF, and ER: N2G26330ZEF (ABRC). Restriction enzyme sites were designed into the primers to allow the specific coding portions from each gene to be ligated in-frame. The PCR-amplified fragments were gel purified and then digested with their respective enzyme (see Supplementary Table 3). The digested fragments were gel-purified then ligated with T4 DNA Ligase and the resulting product was PCR amplified. The products from the final PCR amplification were recombined into the pENTR-D-TOPO vector and verified by sequencing. For example, to generate the KOIN_LRRs-TM_:IRK_KD_ chimera, the *KOIN* fragment was PCR amplified from its ATG through the transmembrane domain with the reverse primer containing an EcoRV cut site such that the 3’ end of this *KOIN* fragment contains a coding region for its extracellular and transmembrane domain, plus a few amino acids that precede the KD. Complementary to this, the forward primer to amplify the IRK KD contains an EcoRV cut site. After amplification and digestion of each with EcoRV, the fragments can be ligated with T4 DNA ligase, thus creating the in-frame KOIN_LRRs-TM_:IRK_KD_ chimera.

### Generation of transgenic plants

Validated expression vectors were transformed into *Agrobacterium tumefaciens* strain GV3101 for transformation into Arabidopsis plants by the floral dip method (Clough and Bent, 1998). Transformed plants with resistance to Basta (dpGreen-BarT) or Kanamycin (dpGreen-KanT) were selected using standard methods. T2 lines exhibiting a resistant-to-sensitive ratio of 3:1, indicating a single insertion locus, were selected for propagation. T3 progeny with 100% resistant seedlings (homozygous) were selected for analysis. At least three independent lines for each reporter showing comparable fluorescence levels and protein distribution profiles were used for subsequent analysis.

Homozygous plants expressing the different constructs were crossed by standard methods. Two independent, representative *pCO2:KOIN:GFP* lines in Col-0 were crossed with *scr-4* heterozygous plants. In the F2 generation, *scr-4* mutant seedlings were identified by their root phenotype and checked by confocal microscopy for KOIN:GFP. Independent lines expressing *SCRp:KOIN-mCh* and *pSCR:IRK:GFP* (each in Col-0) were also reciprocally crossed. Among the F4 progeny, those with 100% Basta and Kanamycin resistant seedlings showing IRK and KOIN fluorescence at comparable levels were selected for further analyses.

### Chemical treatments

Seedlings were grown on 1× MS solid medium plates sealed with tape for 6 d.p.s. For treatments of up to 6 h, seedlings were transferred to 24-well plates (5–8 seedlings per well) containing 1 mL of 1× MS liquid medium containing 50 μM BFA, 40 μM ES2, 30 μM Wm, 50 μM CHX, 20 μM LatB, 20 μM Oryzalin, combinations of these chemicals, or the solvent control. For 24 h BFA treatments, seedlings were transferred to 1× MS solid medium to avoid precipitation of the chemicals. BFA was added to 1× MS agar medium (cooled to 55 °C) at a final concentration of 50 µM before pouring the plates. Control plants were incubated in media containing equal volumes of solvent. Stock solutions of 40 mM BFA (Sigma-Aldrich), 10 mM ES16^5^, 40 mM ES2^31^, 33 mM Wm (Sigma-Aldrich), 50 mM CHX (Sigma-Aldrich), 20 mM LatB (Sigma-Aldrich) and 50 mM Oryzalin (Sigma-Aldrich) were prepared using DMSO as a solvent. The imaging was carried out in the presence of the liquid media, plus the respective chemicals/solvents. At least 20 plants per condition were analyzed. Each treatment was replicated at least three times to assure reproducibility.

### Confocal microscopy and image analysis

Roots were stained with ~10 μM PI solubilized in water for 1–2 min before visualization. Roots were stained with 10 μM FM4-64 diluted in liquid MS media for 5–10 min, to allow sufficient dye penetration to visualize the PM of endodermal cells.

Imaging was performed on a Leica SP8 upright confocal microscope equipped with a water-corrected 40× (1.1 numerical aperture (NA)) objective. Images were generated using PMT and HYD detectors with the pinholes adjusted to 1 Airy unit for each wavelength and system settings as follows: GFP excitation 488 nm, emission at 492–530 nm; YFP excitation 514 nm, emission at 515–550 nm; mCh or mSC excitation 594 nm, emission at 585–660 nm, PI excitation 536 nm, emission at 585–660 nm, and FM4-64 excitation 488 nm, emission at 600–660 nm. In-line sequential scanning was performed when GFP and mCh/mSC or FM4-64 fluorescence was collected in the same root to avoid signal bleed through. Image processing was performed using the LAS X software or ImageJ software (imagej.nih.gov/ij/). For FRAP experiments, image acquisition was performed in the Zeiss LSM 880 inverted microscope using a 40× (1.1 NA) water immersion objective and the ZEN (black edition) software to set the parameters and perform the quantifications.

The two-dimensional graphs displaying IRK-GFP and KOIN-mCh pixel intensities along newly formed PMs during cell divisions were generated with the plot profile plugin in ImageJ. The X-axis represents the distance of the line drawn at the membrane and the *y*-axis is the pixel intensity along that line. To compare IRK-GFP or KOIN-GFP distribution with that of FM4-64 at the PM of CEI and CEID, we selected a rectangular area and used the surface plot plugin in ImageJ to produce a three-dimensional graph of pixel intensities. Additionally, the signal intensity of IRK-GFP and KOIN-mCh after ES2 or Wm treatments was quantified in ImageJ using the Measure tool and applying the formula: corrected signal intensity = integrated density  − (area selected × mean fluorescence of background readings). Fluorescence intensity detected in the root meristem just above the QC from control images (control values) was used as a reference to provide a percentage of signal intensity when compared to the same area in the respective treatments.

For each quantification, the raw data was exported, and the graphic output was produced using Prism (Graphpad, San Diego, CA).

### Phenotypic analyses

Seedlings of the various genotypes being compared were grown side-by-side on 0.2× MS agar plates sealed with parafilm. At 5, 6, and/or 7 d.p.s., roots were stained with ~10 μM PI solubilized in water for 1–2 min and visualized via laser scanning confocal microscopy (Leica Sp8). Z-stacks of the root meristem of 10–15 seedlings per genotype were acquired for 2–3 biological replicates. Endodermal LADs were observed by analyzing transverse optical sections with the orthogonal sectioning tool of the LAS X software. The number of endodermal LADs per transverse section from the QC up to 15 cells above the QC were counted. Therefore, the total number of LADs is the sum of these divisions within this region. Periclinal divisions in the endodermis were counted within the range of QC + 120 μm by examining longitudinal and transverse optical sections. Stele area was quantified in ImageJ by tracing a polygon along the inner surface of endodermal cells at 120 μm above the QC and then the polygon area was calculated by the software.

To measure root length, all plates were scanned, and the resulting images were analyzed using Image J software. To measure root meristem size, the number of cortical cells from the QC to the first elongated cell was counted. For each experiment, at least 15 roots were analyzed, and the mean and standard deviation were calculated. The data were exported and analyzed using Excel and the graphic output was produced using Prism (Graphpad).

### Cell division quantification

Seedlings of the various genotypes being compared were grown side-by-side on 0.2× MS agar plates sealed with parafilm for 7 d.p.s. Seedlings were fixed using 4% paraformaldehyde in MTSB (50 mM PIPES, 5 mM EGTA, 5 mM MgSO4, pH 7) for 1 h and washed three times in MTBS. Cells were permeabilized by treatment for 1 h in 10% DMSO, 3% IGEPAL (Sigma) in MTSB (pH 7). Roots were then stained with ~10 μM PI solubilized in water for 5 min and visualized via laser scanning confocal microscopy (Leica Sp8). Z-stacks of the root meristem of 10–15 seedlings per genotype were acquired for three biological replicates. From the QC up to 250 µm cortex and endodermal cells in metaphase, anaphase or telophase were scored. The data were exported and analyzed using Excel and the graphic output was produced using Prism (Graphpad).

### RT-qPCR analysis

Total RNA was isolated from 20 whole seedlings per genotype in three independent biological replicates for each *koin* allele and the corresponding WT controls using Qiagen’s RNeasy Plant Mini Kit after 7 d.p.s. on 1× MS agar medium sealed with parafilm. First-strand cDNA was synthesized using RevertAid First Strand cDNA Synthesis Kit and the Oligo(dT)18 primer (Thermo Scientific). qRT-PCR reactions were set up using IQ SYBR Green Supermix (BioRad) and analysis was performed on the CFX Connect Real-Time System. For *KOIN*, three mRNA splicing forms have been described (TAIR10) and, therefore, we used a pair of primers to specifically detect *KOIN.1* and another pair to detect *KOIN.2* and *KOIN.3* (Supplementary Fig. 1A and Supplementary Table 2). Standard curves were performed at least in duplicate. Primer pair efficiency values were calculated for each replicate of the standard curves and the average efficiency was used for subsequent analysis. For each genotype and biological replicate, three technical replicates were included. The expression shown is relative to Col-0 and *KOIN* transcript levels were normalized to *PP2A* (At1g13320)^73^ using the Bio-Rad CFX Manager software 3.1.

### Reporting summary

Further information on research design is available in the Nature Research Reporting Summary linked to this article.

## Supplementary information

Supplementary Information

Reporting Summary

## Data Availability

All data generated or analyzed during this study are included in this published article (and its supplementary information files). Source data are provided with this paper. Additional information and materials generated for and/or reported in this article are available from the corresponding author upon request. Source data are provided with this paper.

## Source data

Source Data
