# Supplementary material for: Distinct mechanisms orchestrate the contra-polarity of IRK and KOIN, two LRR-receptor-kinases controlling root cell division
Source: Nat Commun. 2022 Jan 11;13:235. doi: 10.1038/s41467-021-27913-1 (PMC8752632; doi:10.1038/s41467-021-27913-1)
Supplement: Supplementary file 1 — Supplementary Information [file 41467_2021_27913_MOESM1_ESM.pdf]

Supplementary information

Distinct mechanisms orchestrate the contra-polarity of IRK and KOIN, two LRR-receptor-kinases controlling root cell division

Short Title: IRK and KOIN polarity is established by distinct mechanisms

Cecilia Rodriguez-Furlan, Roya Campos, Jessica N. Toth, and Jaimie M. Van Norman*

Department of Botany and Plant Sciences, Center for Plant Cell Biology, Institute of Integrative Genome Biology, University of California, Riverside, Riverside, CA 92521, USA.

*corresponding and contact author: Jaimie M. Van Norman
jaimie.vannorman@ucr.edu

Supplementary information includes:

Supplementary figures 1-12

Supplementary tables 1-3

Supplementary figures and legends

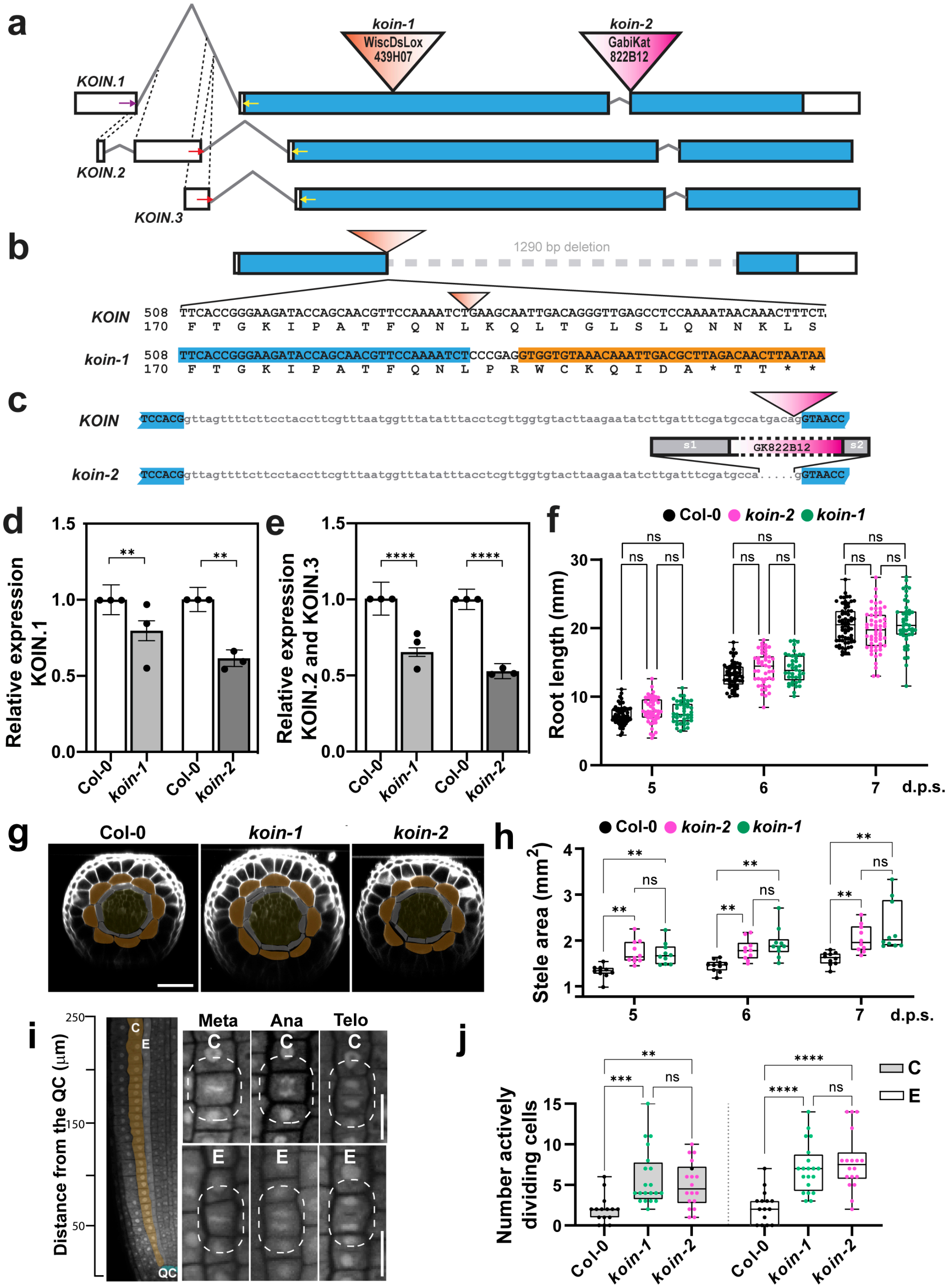

Supplementary Fig. 1. *KOIN* gene structure, mutant alleles, and root phenotypes. (a) *KOIN* (At5g58300) gene models (at 3/16 scale) with introns (gray lines), exons/UTRs (white boxes), and the coding region (blue boxes). The *KOIN* coding region is identical in each splice variant. The T-DNA insertions (not to scale) with intense color indicating the left border. (b, c) KOIN and *koin* genomic regions with numbers at right indicating base pairs (bp, upper) and amino acids (lower). (b) The *koin-1* T-DNA insertion (WiscDsLox_439H07, orange) results in addition of 9 novel amino acids, then premature stop codons (*). Additionally, there is a 1290 bp genomic deletion (gray dotted line) downstream of the insertion. (c) The *koin-2* T-DNA insertion (Gabi-Kat822B12, pink) occurs within the last intron (lower case grey letters) just before the exon begins (blue, uppercase letters). The T-DNA insertion is flanked on each side by novel sequence (gray boxes at ¾ scale, s1 (65 bp) and s2 (21 bp)). (d, e) Expression of *KOIN* isoforms in *koin* mutants relative WT and normalized to the control *PP2A*. Graphs show the average of three independent biological replicates (RNA extracted from 20 seedlings/genotype/replicate) with bars indicating SEM and dots showing data from individual replicates (see Source Data for raw data). Stars indicate statistical significance (** is P value of < 0.01) calculated by Student's T-test. Arrows in (a) indicate the primer locations (not to scale, see Table S2). The forward primers (purple and red) span the exon-exon junction and pair with the same reverse primer (yellow). (f) Root length of WT (Col-0) and *koin* alleles (40-46 roots/genotype) over time (5-7 d.p.s.). (g) Confocal images of transverse sections of root meristems with endodermis (gray) and cortex (orange) highlighted. (h) Quantification of stele area in these genotypes over time 5-7 d.p.s. with bars indicating min./max. values. Data shown from a single representative biological replicate (of three) each containing 15-20 roots/genotype. (i) Confocal image of a WT root fixed at 7 d.p.s. showing the QC (cyan), cortex (orange), and endodermal (gray) cells at various cell cycle stages: metaphase (meta), anaphase (ana), and telophase (telo). (j) Quantification of cells in active stages of cell division in WT, *koin-1*, and *koin-2* (20 roots/genotype) counted from the QC 250 µm up. Scale bars: (g) 25 µm, (i) 10 µm. Abbreviations: ns= no significant difference. In the box plots in f, h, and j whiskers indicate variability outside the upper and lower quartiles

(max/min values), within the boxes median values are indicated by the line and single values are represented as dots, and 1-4 stars indicate the statistical significance ($P < 0.05$ calculated by two-way ANOVA and Tukey’s multiple comparisons test).

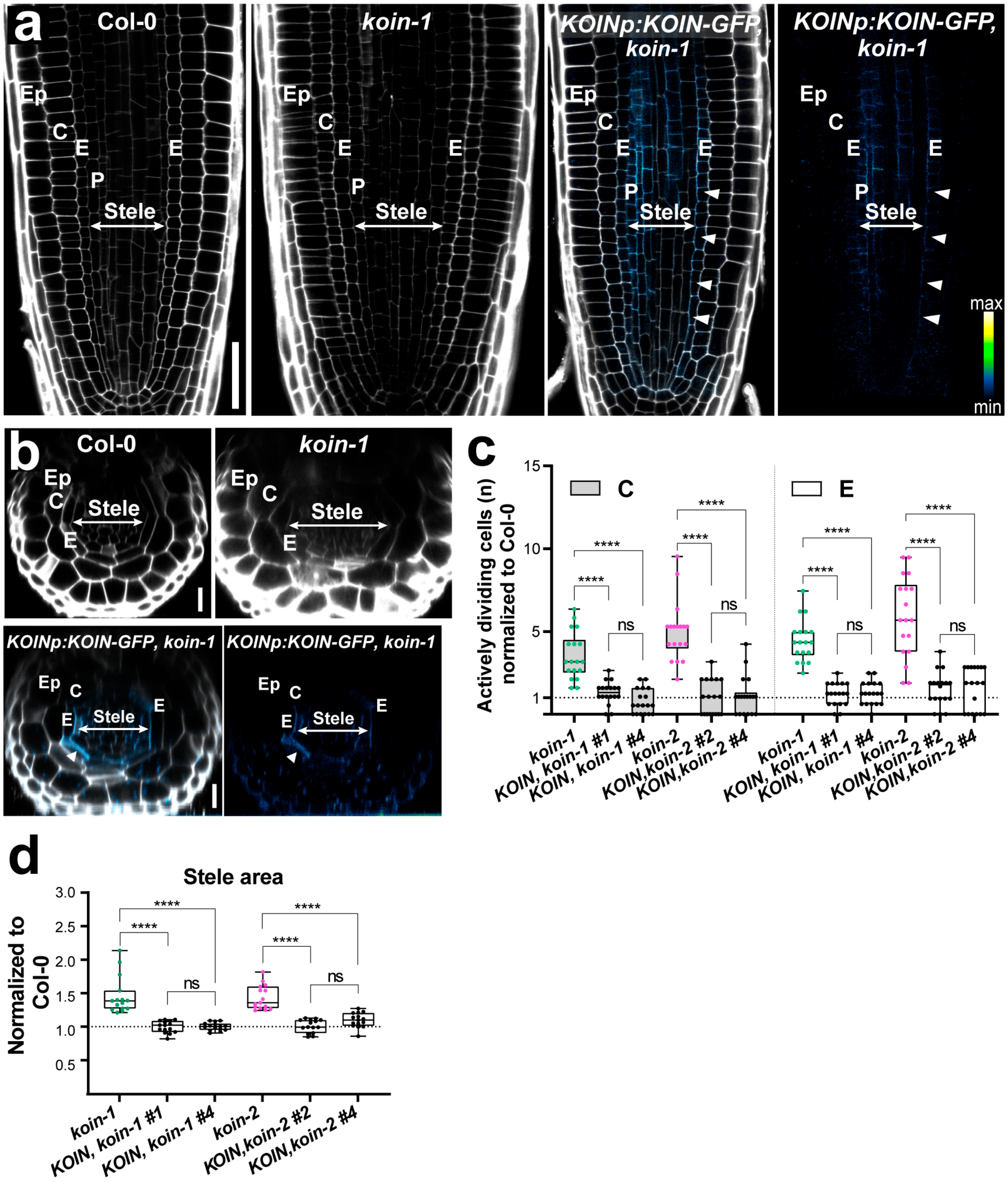

Supplementary Fig. 2. *koin r*oot meristems are larger with increased cell division. Confocal images of the (a) median longitudinal and (b) transverse sections of root meristems expressing *pKOIN:KOIN:GFP #4* at 6 d.p.s. Adjacent panels show merged images of green fluorescent protein (GFP, fluorescence intensity color scale) and PI stain (gray scale) followed by GFP alone. (c) Quantification of endodermal and cortex cells in active stages of cell division and (d) stele area in

koin-1 and *koin-2* and those alleles expressing *pKOIN:KOIN:GFP* with two independent lines each normalized to Col-0. (c-d) Statistical significance 1-4 stars the ($P < 0.05$ calculated by two-way ANOVA and Tukey's multiple comparisons test). Data from a single biological replicate that is representative (of three) with similar results (7 d.p.s. n = 20/genotype). The statistical significance 1-4 stars ($P < 0.05$ calculated by ANOVA and Dunnett's multiple comparisons test). Scale bars: (a) 30 μm, (b) 10 μm.

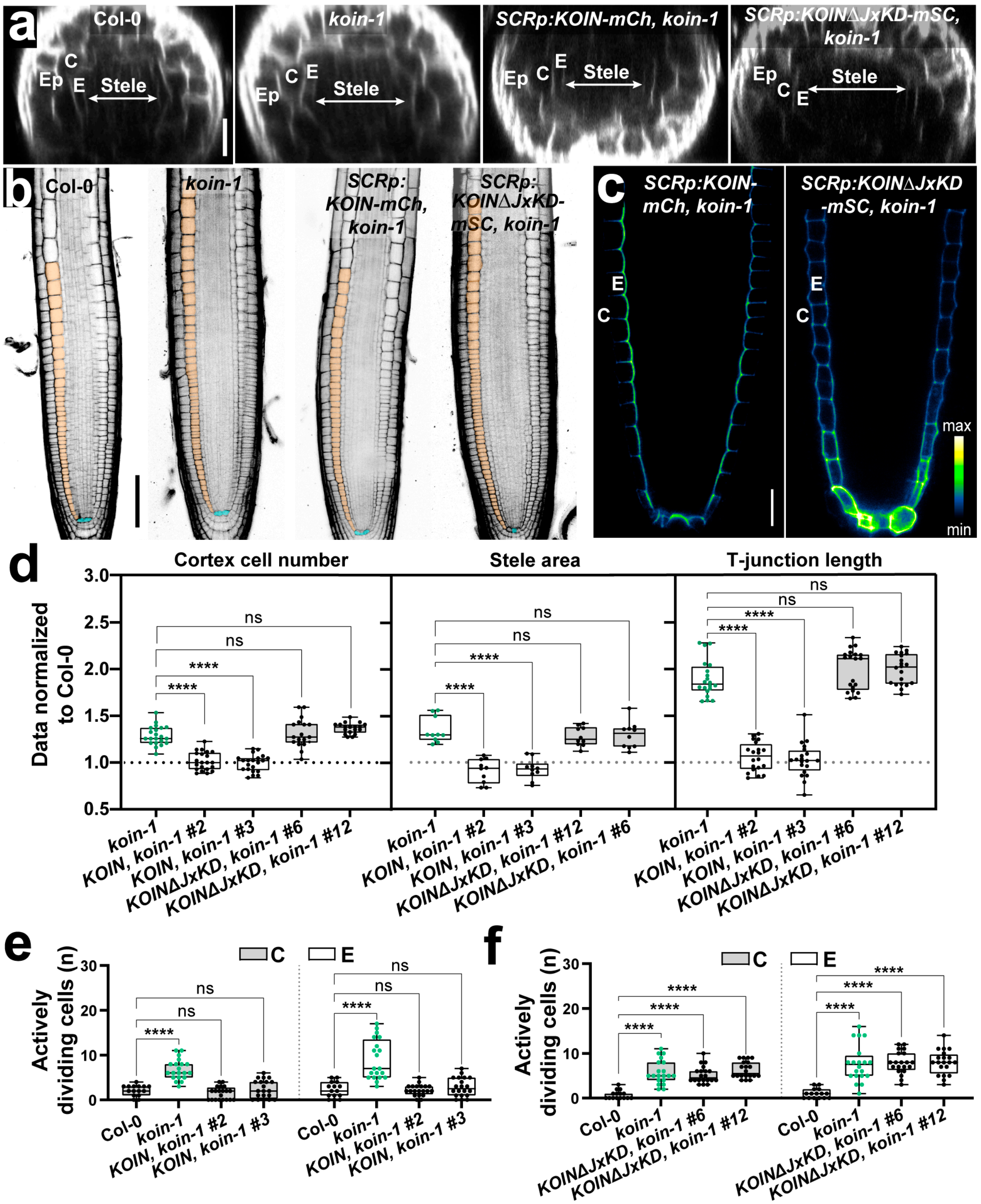

Supplementary Fig. 3: Endodermal expressed KOIN is polarized and rescues *koin-1* mutant phenotypes, whereas KOINΔJxKD is nonpolar and does not rescue. (a, b) Confocal images of transverse (a) and median longitudinal (b) optical sections from root tips of WT, *koin-1* and *koin-1* expressing *SCRp* driven KOIN-GFP and KOINΔJxKD-GFP. Roots stained with PI (a) gray and (b) B/W with meristematic

cortex cells colored in orange and the QC in cyan. (c) Confocal images of median longitudinal sections from *koin-1* root tips expressing *SCRp* driven KOIN-mCHERRY (mCH) and KOINΔJxKD-mSCARLET (mSC) with fluorescence shown in intensity color scale. (d-f) Quantification of various root phenotypes in *koin-1* and *koin-1* expressing *SCRp*:KOIN-mCH or KOINΔJxKD-mSC (two independent transgenic lines each) normalized to Col-0. Graphs show representative results of experiments performed in ≥3 independent replicates, with n = 20 roots/genotype, bars indicate max-min values, and ****= $p < 0.0001$, ns = not significant (two-way ANOVA and Dunett's multiple comparisons test). Whiskers indicate max-min values, in the boxes median values are indicated with a line, and single values are represented as dots Abbreviations: Ep, epidermis, C, cortex; E, endodermis. Scale bars: (a) 20 μm, (b) 50 μm, and (c) 10 μm.

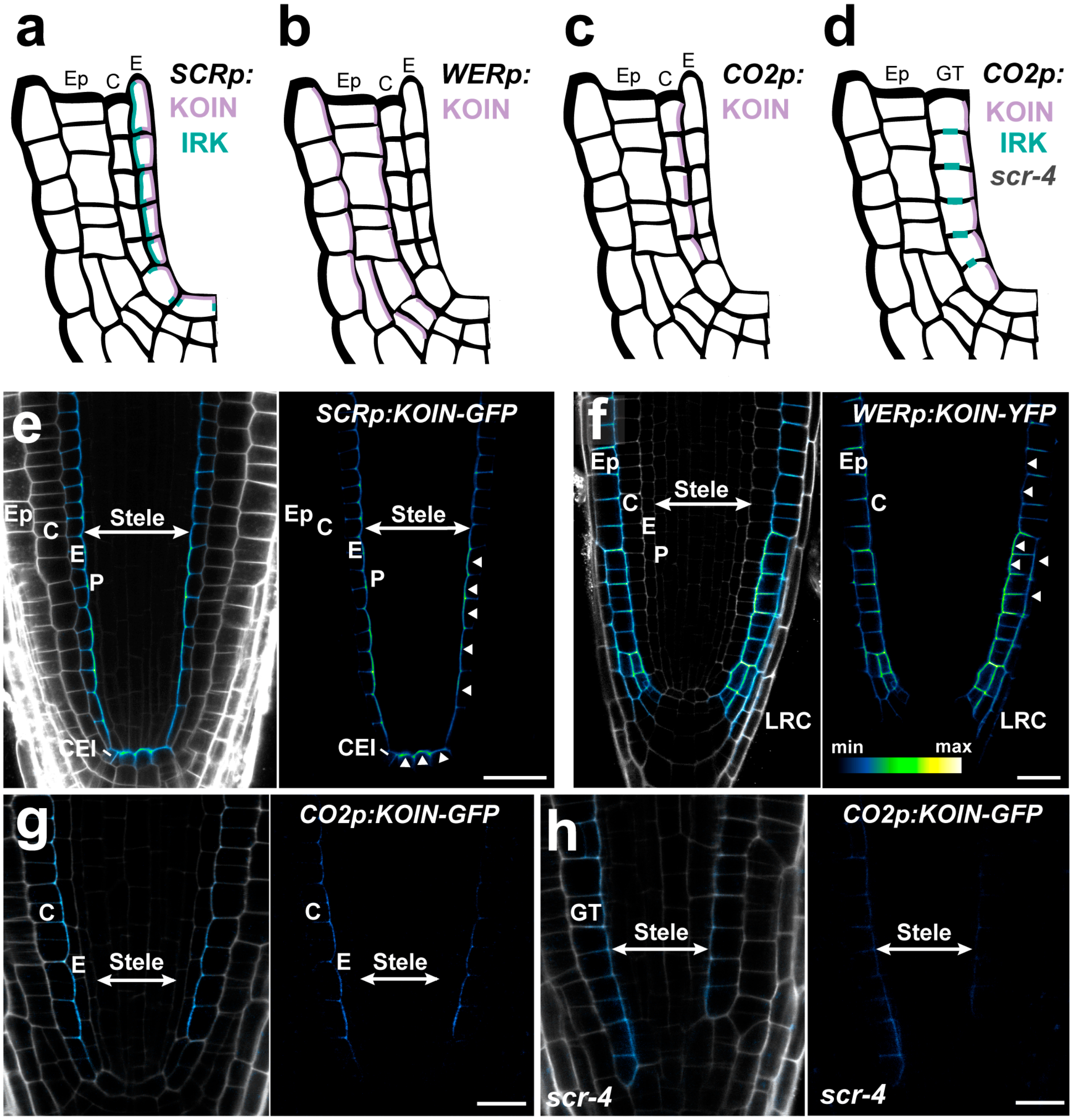

Supplementary Fig. 4: KOIN-GFP is localized to the inner lateral domain regardless of cell type. (a-d) Schematics of a portion of the Arabidopsis root tip showing KOIN and (a, d) IRK localization upon cell layer-specific misexpression in Col-0 (a-c) and scr-4 (d). (e-h) Confocal images of the median longitudinal sections of (e-g) WT and (h) *scr-4* root meristems (at 6 d.p.s.) expressing KOIN-GFP under cell layer-specific promoters (a, e) *SCRp*, (b, f) *WERp*, (c, d, g, h) *CO2p*. Adjacent panels show merged images of green fluorescent protein (GFP, fluorescence

intensity color scale) and PI stain (gray scale) followed by GFP alone. Scale bars: (e, f) 20 µm, (g, h) 10 µm. Images are representative of the results obtained in experiments performed 3 times where at least 15 plants/condition were examined per replicate.

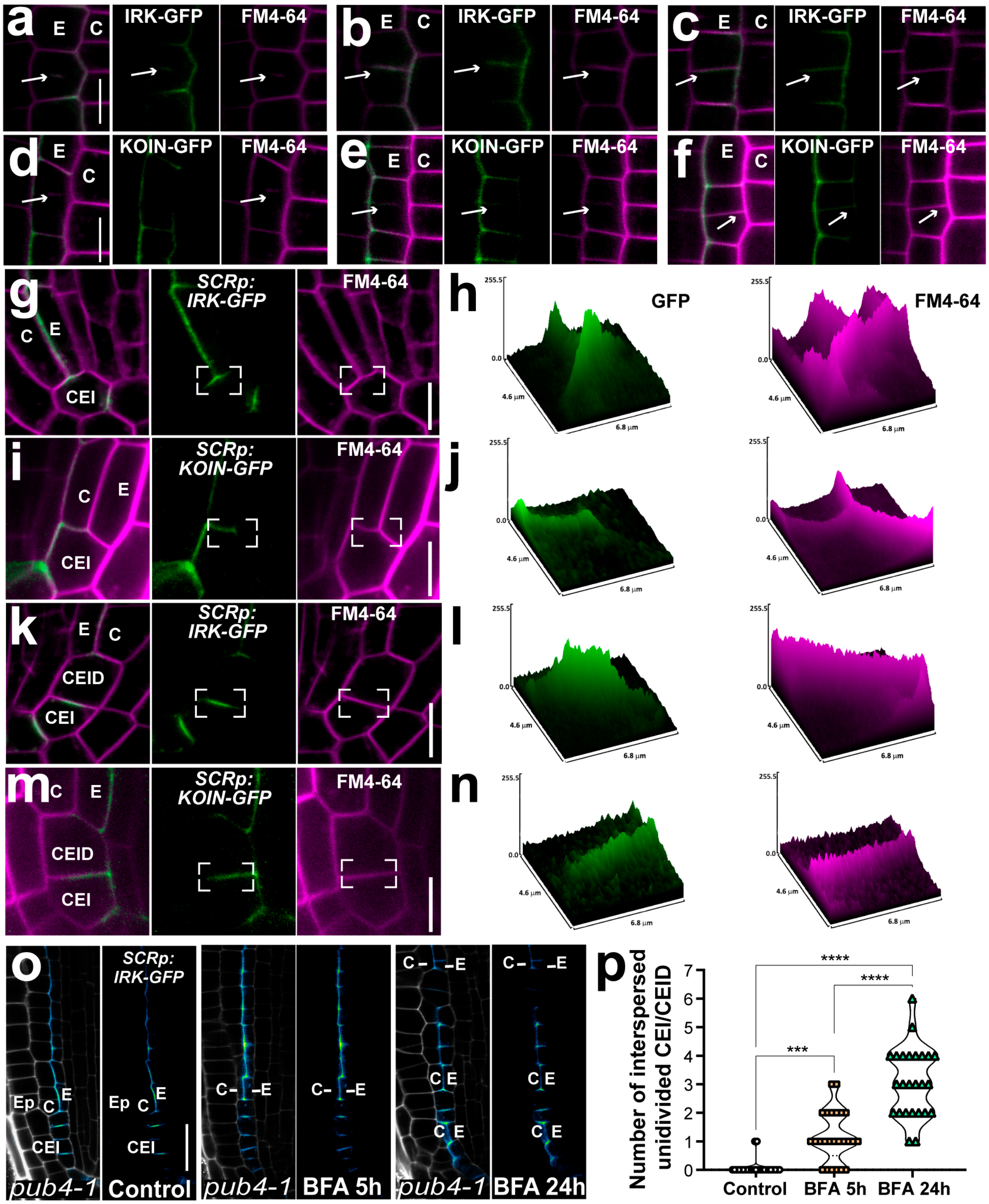

Supplementary Fig. 5: IRK and KOIN are differentially distributed in new cell membranes during proliferative and formative cell divisions. (a-f, g, i, k, m) Confocal images of cells from roots expressing *SCRp:IRK-GFP* or *SCRp:KOIN-GFP* and pulse labeled with FM4-64 for 15 min. (h, j, l, n) Surface plots of linear fluorescence intensities for IRK-GFP or KOIN-GFP (green) and FM4-64 (magneta)

during various formative cell divisions in boxed regions in (g, k, i, m). (a-c) IRK-GFP or (d-f) KOIN-GFP and FM4-64 distribution at different stages of cell plate formation during proliferative (transverse anticlinal) cell divisions. Distribution of (g) IRK-GFP or (i) KOIN-GFP at the shootward membrane of the CEI. (h) Surface plot showing IRK-GFP and FM4-64 at the intersection between CEI-Endodermis-Cortex cells and (j) KOIN-GFP accumulation at the shootward face of the CEI in contact with the cortex cell. (k-l) IRK-GFP centralized accumulation at the membranes between the CEI and CEID while FM4-64 is evenly distributed. (m-n) KOIN-GFP distribution at the membranes between the CEI and CEID with higher signal towards the inner membranes, while FM4-64 signal is evenly distributed. (o) Confocal images of *pub4-1* root tips expressing *SCRp:IRK-GFP* (GFP with fluorescent intensity color scale) and stained with PI (gray) merged and GFP alone. (Left to right) *pub4-1* root tips under control conditions (DMSO) and treated with 50 µM BFA for 5 h and 24 h. (p) Quantification of the number of CEI/CEID cells that are interspersed with cells that have already divided to form endodermis and cortex in *pub4-1* under these conditions (25 roots/condition). Significance 1-4 stars $P < 0.05$ calculated by one-way ANOVA using Turkey´s multiple comparison test. Scale bars: 5 µm, except (o) 20 µm. Abbreviations: C, cortex; E, endodermis; CEI, cortex/endodermis initial; CEID, CEI daughter. Images displayed are representative of experiments replicated at least 3 times with each replicate containing at least 15 plants/condition.

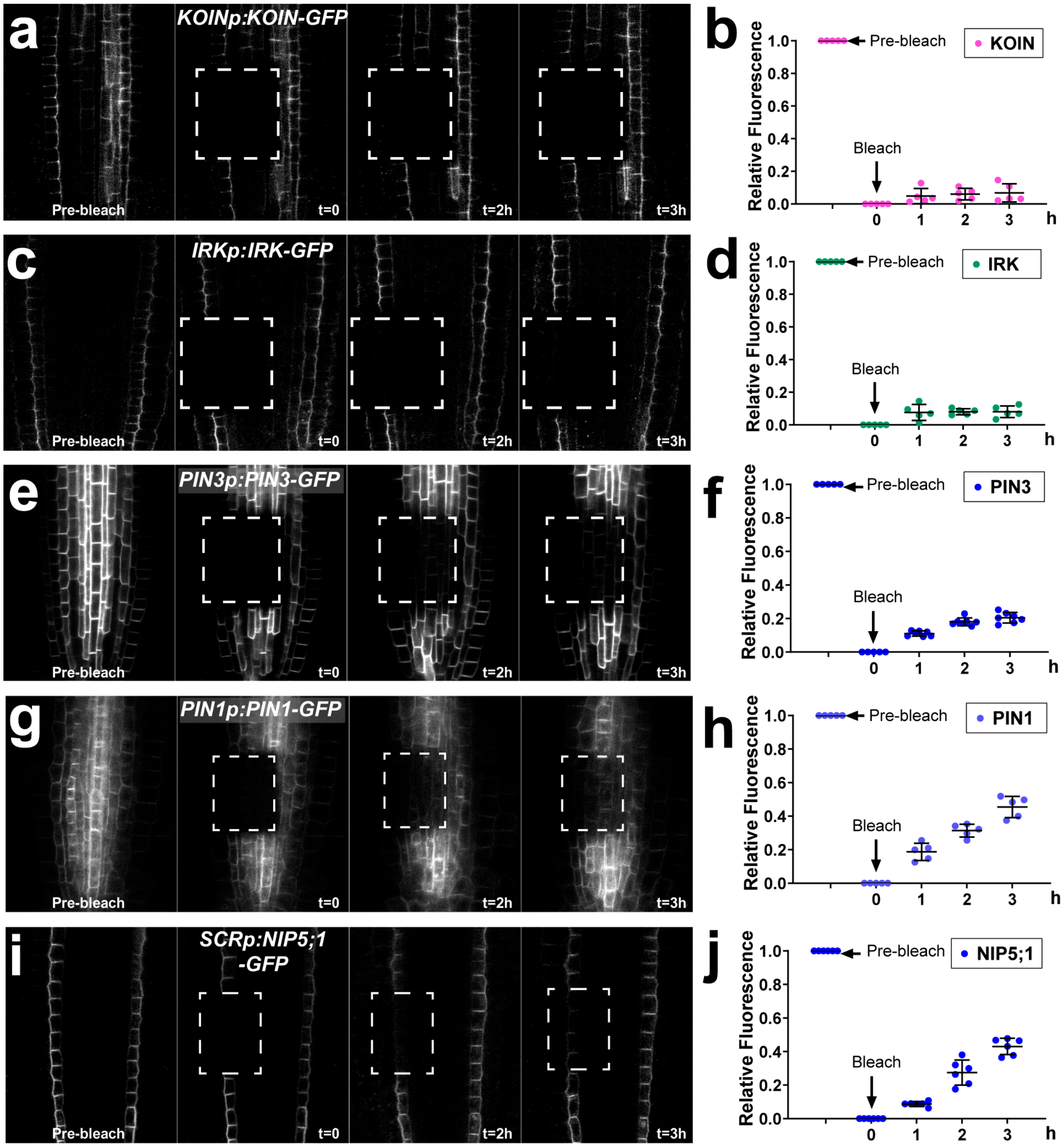

Supplementary Fig. 6: IRK-GFP and KOIN-GFP show little recovery at the PM after photobleaching. Representative images from FRAP experiments of endogenously expressed (a) IRK-GFP, (c) KOIN-GFP, (e) PIN3-GFP, (g) PIN1-GFP, and (i) *SCRp* expressed NIP5;1-GFP. (Left to right) images of root pre-bleach, then immediately after bleaching (t=0) and 1 and 2 hours after with photobleached area indicated by dashed square. (b, d, f, h, j) Dot plots showing relative fluorescent signal

recovery (with t=0 set to zero and pre-bleach set to 1) of 15 roots/genotype. Bars represent mean with SD values.

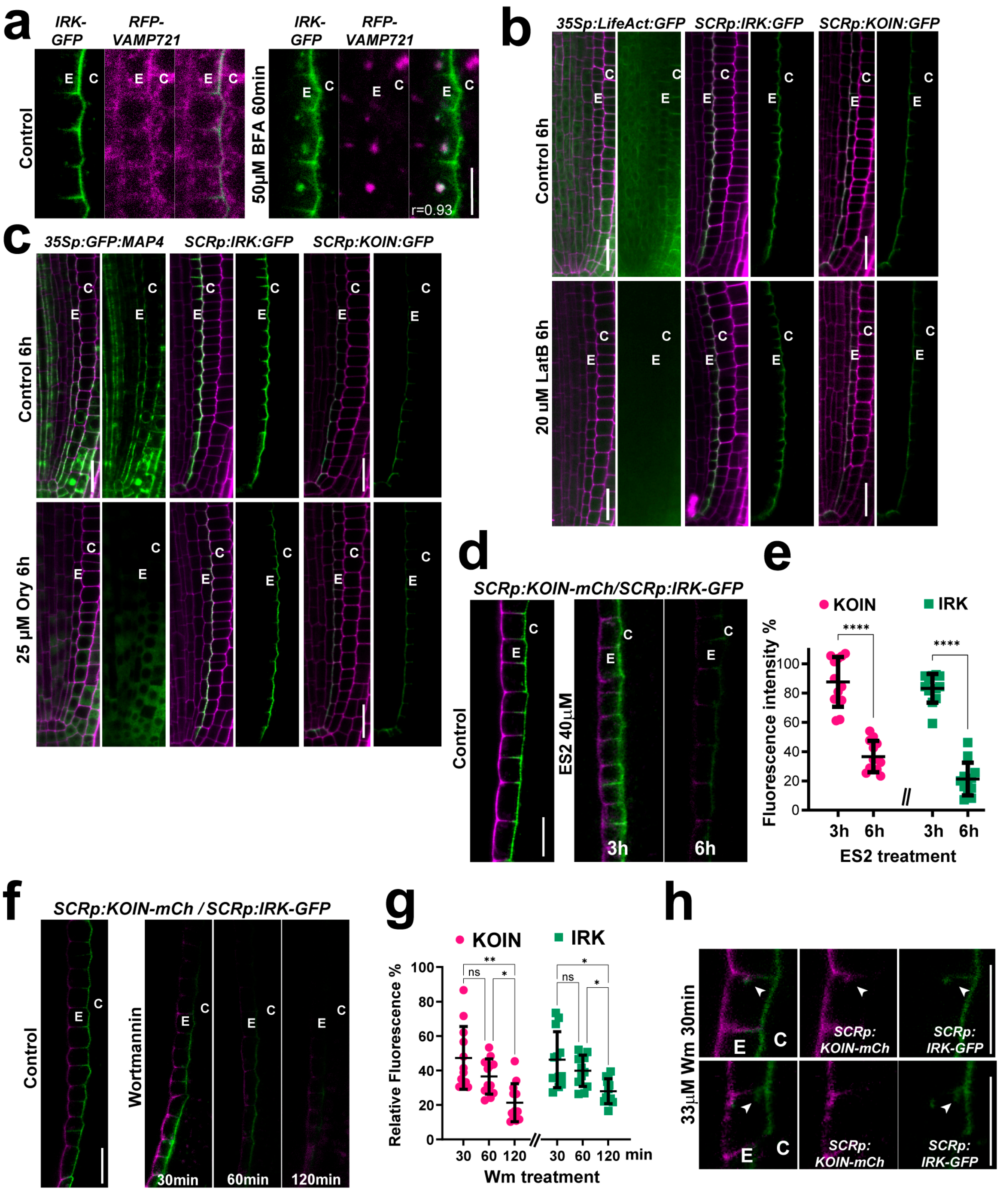

Supplementary Fig. 7: Chemical treatments in IRK-GFP and/or KOIN-GFP expressing roots. (a) Consecutive panels show confocal images of root tissues

expressing *SCRp:IRK-GFP* (green) and RFP-VAMP721 (magenta) and the merged images under controls and BFA treatment. Upon BFA treatment IRK-GFP and RFP-VAMP721 colocalize with a Pearson correlation coefficient of $r=0.93$. (b) Consecutive panels show confocal images of F-actin (LifeAct-GFP, green), *SCRp:IRK-GFP* or *SCRp:KOIN-GFP* (green) with PI counterstain (magenta) during 6h treatment with DMSO (control) or the actin depolymerizing drug Latrunculin B (LatB). (c) Consecutive panels show confocal images of microtubule marker (35S:GFP-MAP4, green), *SCRp:IRK-GFP,* and *SCRp:KOIN-GFP* (green) in root cells with PI counterstain (magenta) during 6 h treatment with DMSO (control) or the microtubule disrupting drug Oryzalin (Ory). (d) Roots expressing *SCRp* driven IRK-GFP and KOIN-mCh treated with 40 µM ES2 for 3 h and 6 h. (e) Graph of IRK-GFP and KOIN-mCh signal intensity at the PM in ES2-treated endodermal cells. The mean values of the signal intensities of 20 roots/time point were normalized to those of 20 control roots (expressed as percentage, %). Graph and images show representative results of experiments replicated 3 times with $n = 20$ plants/condition, bars represent the SD values and individual values are displayed. **** = $p < 0.0001$ calculated by two-way ANOVA. (f) Accumulation of *SCRp:IRK-GFP* (green), *SCRp:KOIN-mCh* (magenta) at the PM in control conditions or after treatment with 33 µM Wortmannin (Wm), a drug that inhibits the synthesis of phosphoinositols (PI4P, PI3P). (g) Quantification of the fluorescence intensity of IRK-GFP (green) and KOIN-mCh (magenta) after 30, 60 and 120 min of Wm treatment expressed as a percentage relative to control conditions (graphs and images show representative results of experiments replicated three times with $n = 12$ plants/condition). Bars represent the SD and individual values are shown. ** = $p < 0.001$, * = $p < 0.05$, ns = not significant calculated by two-way ANOVA. (h) Intracellular accumulation of IRK-GFP (green) or KOIN-mCh (magenta) in endodermal cells after 30 min Wm treatment. The arrowhead shows co-localization or independent localization of IRK-GFP and KOIN-mCh in doughnut shape structures. Scale bars: (a, d, f, h) 10 µm and (b, c) 20 µm.

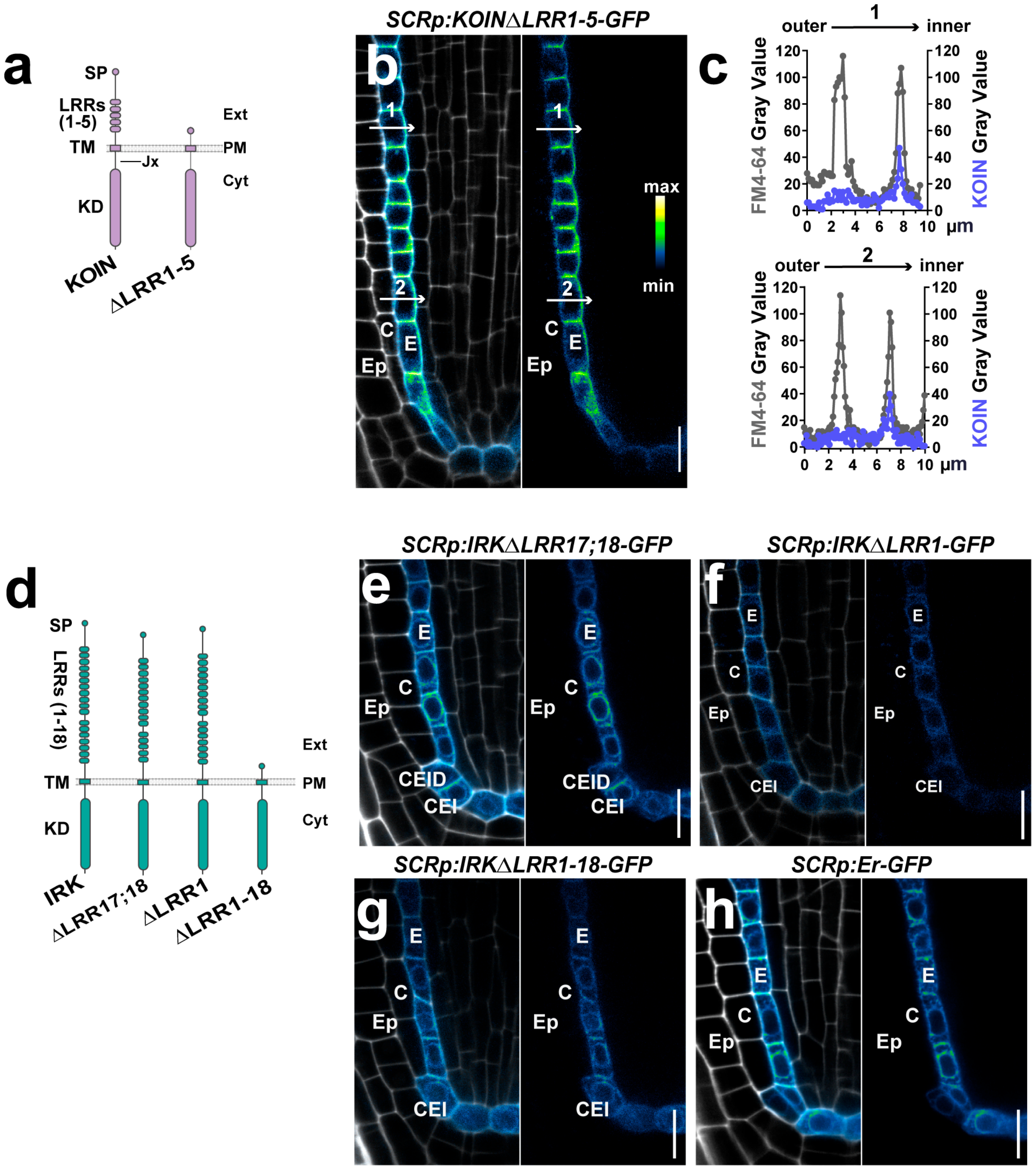

Supplementary Figure 8: Deletion of KOIN and IRK LRR domains differentially impacts protein accumulation at the plasma membrane. (a) Schematics of KOIN domain structure (purple) and with the LRR domains deleted (KOINΔLRR1-5). (b) Adjacent panels show confocal images of endodermal (*SCRp*) expressed KOINΔLRR1-5-GFP signal (intensity false colored) in roots counterstained with FM4-64 (gray) merged and GFP alone. (c) Quantification of the fluorescence

intensity of KOINΔLRR1-5-GFP (purple) and FM4-64 (gray) across two endodermal cells marked 1 and 2 (white arrows). The graphs show that only in the inner lateral face has a peak of KOINΔLRR-GFP fluorescence that coincides with the peak of FM4-64 labeling the PM. This indicates that the proportion of protein that is not retained in the ER (basal intracellular GFP signal) localizes to the inner PM. (d) Schematics of IRK domain structure (green) and with LRR deletions: LRR domain 17-18 (IRKΔLRR17-18); LRR domain 1 (IRKΔLRR1), and all LRR domains (IRKΔLRR1-18). (e-h) Adjacent panels show confocal images of roots with GFP signal (signal intensity false colored) and counterstained with PI (gray) merged and GFP alone. Endodermal (*SCRp*) expressed IRKΔLRRs all show intracellular distribution that coincides with (h) *SCRp* driven endoplasmic reticulum-localized GFP (ER-GFP), which directs GFP to remain in that organelle. Abbreviations: signal peptide (SP), transmembrane domain (TM), juxtamembrane domain (Jx), kinase domain (KD), epidermis (Ep), cortex (c), endodermis (e), cortex/endodermis initial (CEI), and CEI daughter (CEID). Scale bars: 10 µm. Images displayed in this figure are representative of observations made in at least 20 plants/genotype and for each genotype at least 3 independent transgenic lines were analyzed.

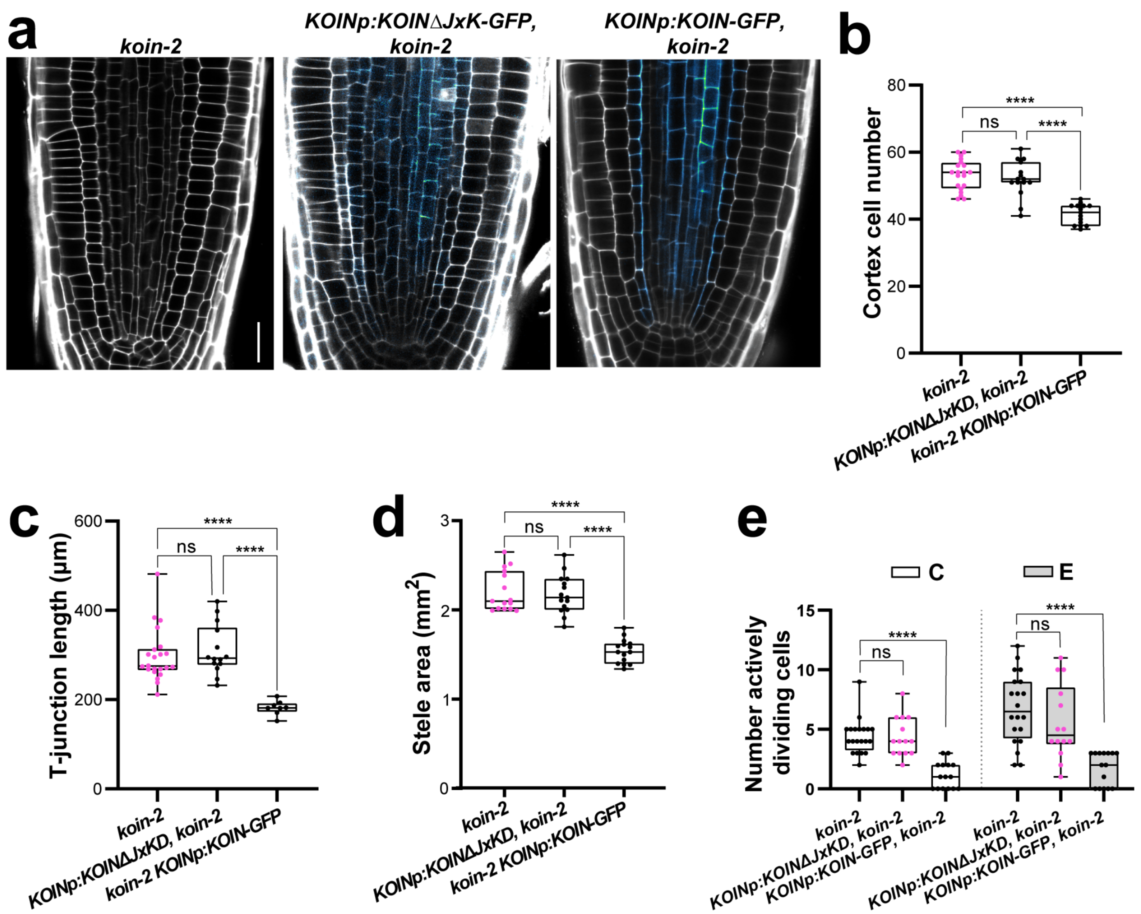

Supplementary Figure 9: Endogenously expressed KOIN truncation, KOINΔJxKD, had non-polar localization and does not rescue *koin* phenotypes. (a) Confocal images of median longitudinal optical sections from root tips of *koin-2* and *koin-2* expressing *KOINp* driven KOINΔJxKD-GFP and KOIN-GFP. Merged images of roots stained with PI (gray) and GFP with intensity color scale. Scale bar: 20 µm. (b, c, d) Quantification of cortex cell number, T-junction length, and stele area in these genotypes with bars indicating min./max. values, in the boxes median values are indicated with a line, and single values are represented as dots. Data shown from a single representative biological replicate (of three) each containing 15-20 roots/genotype. Abbreviations: ns = no significant difference and 1-4 stars the statistical significance ($P < 0.05$ calculated by one-way ANOVA using Dunnett's multiple comparation test). (e) Quantification of cells in active stages of cell division in *koin-2*, and *koin-2* expressing *KOINp* driven KOINΔJxKD-GFP and KOIN-GFP (20 roots/genotype) counted from the QC 250 µm upwards. 1-4 stars the statistical

significance (* is P < 0.05 calculated by two-way ANOVA and Tukey's multiple comparisons test).

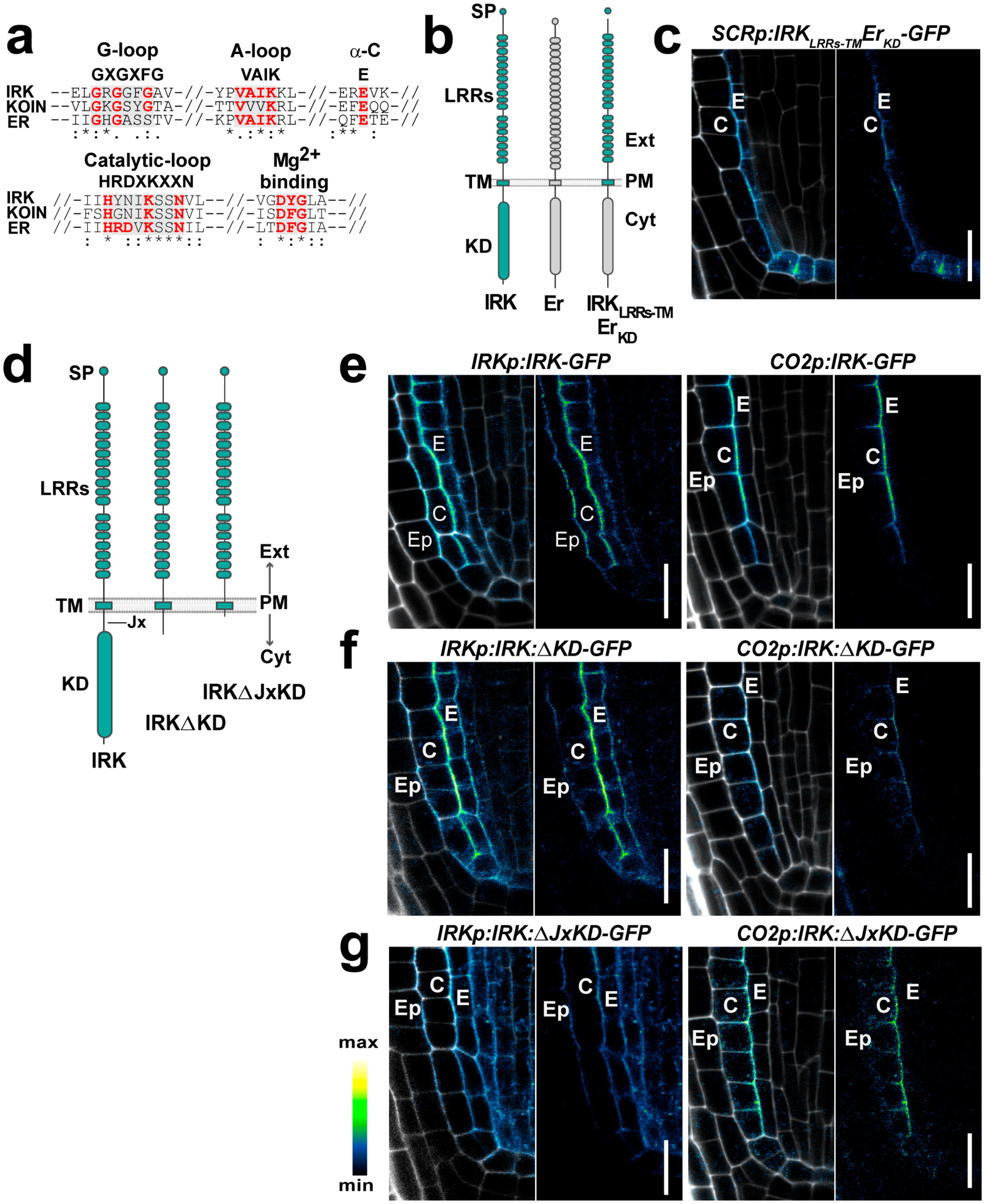

Supplementary Figure 10: The presence of the IRK extracellular domain is sufficient for polar protein accumulation at the plasma membrane. (a) Amino

acid alignment of regions of the IRK, KOIN, and ERECTA kinase domains showing key functional motifs (gray boxes) that participate in ATP binding or hydrolysis. Key residues are indicated (red) in the glycine-rich G-loop, the adenine binding A-loop, the putative conserved glutamic acid-E in the alpha C-helix (α-C), the catalytic loop, and the Mg2+ ion-binding motif. IRK and KOIN, atypical kinases, have non-conserved residues in the catalytic loop, whereas active kinases, like ERECTA, have the conserved residues in the catalytic loop. (b) Protein structure diagrams of IRK (green), ERECTA (gray), and the IRK/ERECTA chimera. (c) Confocal images showing *SCRp* expressed $IRK_{LRRs\text{-}TM}ERECTA_{KD}$-GFP distribution in the root tip with adjacent panels showing GFP+PI merged or GFP alone. (d) Protein structure diagrams of IRK and truncated IRK lacking the kinase domain (IRKΔKD) or the juxtamembrane (Jx) and kinase domain (IRKΔJxKD). (e-g) Paired confocal images show GFP signal (intensity color scale) and the merged images with GFP+PI counterstaining (gray). Proteins expressed under *IRKp* or cortex-specific *CO2p*. Localization of (e) full-length IRK-GFP, (f) IRKΔKD-GFP and (g) IRKΔJxKD-GFP. Scale bars: 10 µm. Abbreviations: signal peptide (SP), transmembrane domain (TM), kinase domain (KD), extracellular (Ext), PM (PM), cytoplasm (Cyt), Ep, epidermis, C, cortex; and E, endodermis. Confocal images displayed are representative of observations made in at least 20 plants/genotype and for each genotype at least 3 independent transgenic lines were analyzed.

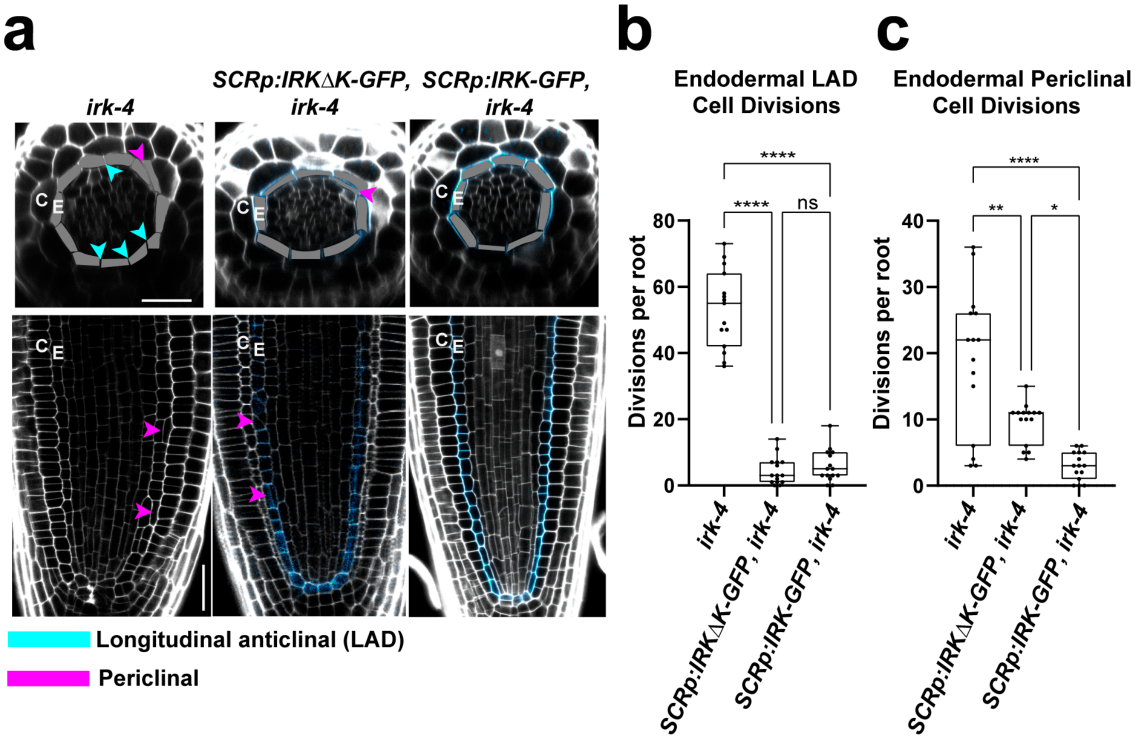

Supplementary Figure 11. Endodermal expressed IRK truncation, IRKΔKD, is polarized and partially rescues *irk-4* phenotypes. (a) Confocal images of transverse and median longitudinal optical sections from root tips of *irk-4* and *irk-4* expressing *SCRp* driven IRKΔKD-GFP or IRK-GFP. Merged images of roots stained with PI (gray) and GFP with intensity color scale with endodermal longitudinal anticlinal (cyan arrowheads) and periclinal (magenta arrowheads) cell divisions indicated and endodermal cells highlighted in upper panels. Quantification of endodermal (b) longitudinal anticlinal and (c) periclinal cell divisions (n = 20 roots/genotype). Graphs b and c show representative results of experiments performed in ≥ 3 independent replicates, bars indicate max/min values and 1-4 stars statistical significance P value < 0.05 (*), ns = not significant (two-way ANOVA and Tukey's multiple comparisons test). Scale bars: 20 μm. Abbreviations: cortex (c), endodermis (e).

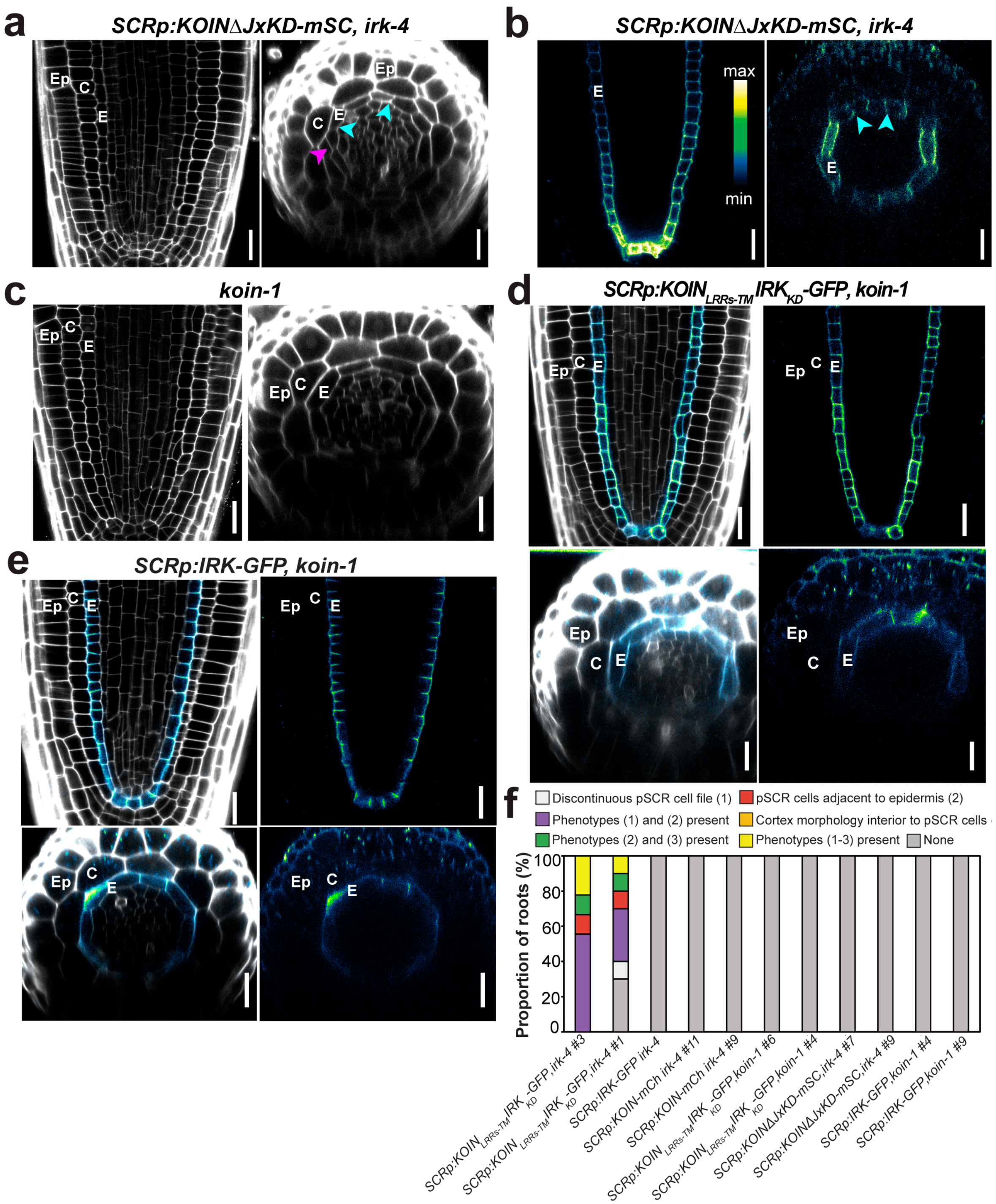

Supplementary Fig. 12. No additional cell division phenotypes are observed upon endodermal expression of the KOIN/IRK-GFP chimera in *koin*. (a-e) Confocal images of median longitudinal and transverse sections of (a, b) *irk-4* and (c-e) *koin-1* mutant roots stained with PI (gray) and/or fluorescence (intensity false colored). (a, b) *irk-4* roots expressing *SCRp:KOINΔJxKD-mSC* (a) stained with PI

(gray) and showing (b) KOINΔJxKD-mSC localization with endodermal longitudinal anticlinal (cyan arrowheads) and periclinal (magenta arrowheads) cell divisions. (C-E) *koin-1* and *koin-1* roots expressing *SCRp:KOIN*$_{LRRs\text{-}TM}$*IRK*$_{KD}$*-GFP* and *SCRp:IRK:GFP*. (f) Quantification of roots exhibiting any or all of the enhanced cell division phenotypes (see also Figure 6): (1) discontinuous cell files or rings expressing *SCRp*, (2) cells expressing *SCRp* immediately adjacent to the epidermis, and/or (3) cells with cortex morphology interior to cells expressing *SCRp* in various genotypes. Note these phenotypes are only present in *irk-4* expressing this chimera. Data shown from a single representative biological replicate (n = 9-10 roots/genotype). Abbreviations: (Ep) epidermis; (c) cortex; (e) endodermis. Scale bar= 20 µm.

Supplementary Table 1: Summary of chemical treatments to examine protein trafficking contribution to KOIN and IRK polarity.

Small molecule	Molecular and cellular target	Treatment Conditions	Consequences/Visualization	Effect on IRK and KOIN trafficking	Conclusions
**Brefeldin A (BFA)**	Binds the SEC7 domain of a subset of the GEFs, causing the inhibition of Golgi and post-Golgi trafficking that affects delivery to the PM of a subset of de novo synthesized and recycled proteins.	**50 µM 1 h**	Agglomerations of Golgi and TGN compartments. [1,2]	IRK, but not KOIN, is observed in BFA bodies in the endodermis.	IRK, but not KOIN, is secreted by a pathway partially sensitive to BFA.
		**50 µM 4-5 h**	Cell plate formation is disrupted, delays in new PM and cell wall positioning[1].	IRK, but not KOIN, is agglomerated near the new PMs after cell division affecting IRK accumulation.	During cell division, IRK but not KOIN, is secreted to the new PM via a BFA sensitive pathway.
		**50 µM 24 h**	Cell divisions eventually occur, but with some altered cell division plane orientation and numerous binucleated cells [1].	IRK is observed in newly formed PM after CEIDs have divided.	BFA delays CEID division in *pub4-1* allowing visualization of IRK lateral polarity in endodermal cells.
**Cycloheximide (CHX)**	Binds the 60S ribosome tRNA E-site inhibiting protein synthesis	**50 µM 2 h**	Decreased production of newly synthesized protein [3].	IRK and KOIN accumulation at the PM decreases.	IRK and KOIN accumulation at the PM is affected by protein synthesis inhibition.
**CHX+ (CHX+BFA)**	Inhibition of protein synthesis prior to BFA inhibition of protein secretion.	**50 µM CHX 1 h + (50 µM CHX + 50 µM BFA) 1 h**	BFA agglomerations now contain mainly proteins internalized from the PM because of the in inhibition of secretion back to the PM (recycling) [4].	IRK is no longer observed in BFA agglomerations.	IRK agglomeration in BFA bodies corresponds to newly synthesized protein.
**Endosidin 16 (ES16)**	Binds RABA2A causing inhibition of exocytosis for a subset of newly synthesized and recycling proteins.	**ES16 15 µM 3 h**	Agglomeration of Golgi and TGN compartments [5].	KOIN, but not IRK, is observed in ES16 agglomerations.	KOIN, but not IRK, is secreted via a pathway sensitive to ES16.
**Endosidin 2 (ES2)**	Binds the EXOCYST subunit EXO70A1 inhibiting exocytosis of a subset of newly synthesized and recycled proteins.	**ES2 40 µM 3 h**	Agglomeration of vesicles near the Golgi and TGN [6].	IRK and KOIN are observed in ES2 agglomerations.	Both IRK and KOIN are secreted by an EXO70A1-dependent mechanism(s).
		**ES2 40 µM 3.5 - 6 h**	PM protein turn-over is visibly affected due to the re-direction of newly synthetized protein traffic to the vacuole [7].	IRK and KOIN accumulation at the PM is greatly decreased.	Newly synthesized IRK and KOIN are likely degraded.
**Wortmannin (Wm)**	Affects the activity of phosphatidylinositol 3- (PI3K) and 4- (PI4K) kinases decreasing the amount of PtdIns3P and PtdIns4P in cellular membranes.	**Wm 33 µM 30 min-1 h**	PtdIns3P and PtdIns4P depletion affects TGN and post-Golgi traffic towards the vacuole causing homotypic fusion of compartments leading to characteristic doughnut shaped structures [8].	IRK and KOIN are observed in doughnut shaped structures.	IRK and KOIN transit to the vacuole through a Wm-sensitive pathway.
		**Wm 33 µM 2 h**	PtdIns4P depletion also affects EXO70A1 association with the PM interfering with EXOCYST dependent exocytosis [9].	IRK and KOIN accumulation at the PM is greatly reduced.	Both IRK and KOIN secretion to the PM is affected.
**Oryzalin (Ory)**	Binds alpha-tubulin, inhibiting microtubule polymerization	**Ory 25 µM 6 h**	Affects cell division and a subpopulation of the TGN causing small intracellular agglomerations [10].	IRK and KOIN accumulation at the PM remains unchanged.	IRK and KOIN accumulation at the PM is not affected by inhibition of microtubule or actin polymerization.
**Latrunculin B (LatB)**	Sequesters actin monomers, inhibiting actin polymerization.	**LatB 20 µM 6 h**	PIN1 polarization is affected by LatB action [11].		

Supplementary Table 2. Expression vectors generated in this paper.

Expression vector	Backbone
KOINp:KOIN-GFP	dpGreen-BarT
SCRp:KOIN-GFP	dpGreen-BarT
SCRp:KOIN-mCh	dpGreen-KanT
WERp:KOIN-3´WER-YFP	dpGreen-BarT
CO2p:KOIN-GFP	dpGreen-BarT
SCRp: KOIN$_{LRRs\text{-}TM}$IRK$_{KD\text{-}}$GFP	dpGreen-BarT
SCR:KOIN$_{LRRs}$IRK$_{TM\text{-}KD\text{-}}$GFP	dpGreen-BarT
SCRp:IRK$_{LRRs\text{-}TM}$KOIN$_{KD}$-GFP	dpGreen-BarT
SCRpIRK$_{LRRs}$KOIN$_{TM\text{-}KD}$-GFP	dpGreen-BarT
IRK$_{LRRs\text{-}TM}$ER$_{KD}$-GFP	dpGreen-BarT
SCRp:IRKΔKD-GFP	dpGreen-BarT
CO2p:IRKΔKD-GFP	dpGreen-BarT
IRKp:IRKΔKD-GFP	dpGreen-BarT
IRKp:IRKΔJx;KD-GFP	dpGreen-BarT
CO2p:IRKΔJx;KD-GFP	dpGreen-BarT
SCRp: IRKΔJx;KD-GFP	dpGreen-BarT
SCRp:KOINΔJx;KD-mSC	dpGreen-KanT
SCRp:IRKΔLRR17;18-GFP	dpGreen-BarT
SCRp:IRKΔLRR1-GFP	dpGreen-BarT
SCRp:IRKΔLRR1-18-GFP	dpGreen-BarT
SCRp:KOINΔLRRs-GFP	dpGreen-NorfT
SCRp:IRK$_{LRRs\text{-}TM}$KOIN$_{KD}$-GFP	dpGreen-NorfT
KOINp:KOIN-GFP	dpGreen-NorfT
KOINp: KOINΔJx;KD-GFP	dpGreen-BarT

Supplementary Table 3: Primers used in this study.

Primer Purpose	Primer name	Primer sequence (5'-3´)
**Primers to generate reporters**		
IRK promoter region	IRKpro_F	CACAGCCCTTATTCATCTCCTAC
	IRKpro_R	CTTTCCACAACCCTCTTCTCC
IRK coding region	IRKcod_F	CACCATGTACAAAGCACTGATTTTTACAGTC
	IRKcod_R	ACTTGAACCCAACTCATCTGAG
KOIN promoter region	KOINpro_F	AGTGAAGAGAAAGCAGAAGTGGA
	KOINpro_R	CAGCTTGACTTCTTGACCCT
KOIN coding region	KOINcod_F	CACCATGATGCAGTTCCATTTCCAGTT
	KOINcod_R	AACTTGGACGTTGGAGTCCTT
**Primers to generate chimeras**		
KOIN extracellular (LRR and TM)	KOINLRR,TM_EcoRV_codR	TTACGATGATATCTTCCCTCTTGTC
IRK cytoplasmic (kinase only)	IRKK_EcoRV_codF	GTTGGTGTAATAGATATCACGGTAC
KOIN TM and cytoplasmic	KOINTM,K_BamHI_codF1	CACAAAGAAGGATCCAAAAGGAA
IRK extracellular (LRR only)	IRKLRR_BamHI_codR1	CTAAGCAGGATCCTTTGTGAC
KOIN extracellular (LRR only)	KOINLRR_BamHI_codR1	TCCTTTTGGATCCTTCTTTGTGAG
IRK TM and cytoplasmic	IRKTM,K_BamHI_codF1	GGTCACAAAAGGATCCTTGCTT
IRK extracellular (LRR and TM)	IRKLRR,TM_EcoRV_codR1	GACTGAAGATATCTCCGCCTGA
KOIN cytoplasmic (kinase only)	KOINK_EcoRV_codF1	TGCTGCGATATCAAGAAAAAGGAC
IRK extracellular (LRR only) - for ERECTA	IRKLRR_StuI_codR	ATCCTTTTGAGGCCTGCAC
ERECTA TM and cytoplasmic	ERTM,K_StuI_codF	TAGTAGGCCTAAACTCACCGT
IRK extracellular (LRR and TM)-for ERECTA	IRKLRR,TM_BamHI_codR	CTGATCTTGGATCCCGTTGAT
ERECTA cytoplasmic (kinase only)	ERK_BamHI_codF	GATGGATCCCTTGACAAACCA
ERECTA extracellular (LRR only)	ERLRR,TM_SacII_codR	GAGGAGGATTCCGCGGTC
IRK TM cytoplasmic - for ERECTA	IRKTM,K_speI_codF	GGTCACAAAAGGATACTGACTAGT
ERECTA extracellular (LRR and TM)	ERLRR,TM_SacII_codR	GAGGAGGATTCCGCGGTC
	IRKK_SacII_codF	ACGGTACTTAATCCGCGGGT

IRK cytoplasmic (kinase only)-for ERECTA	ER_codF	CACCATGGCTCTGTTTAGAGATATTGTTC
	ER_codR	CTCACTGTTCTGAGAAATAACTTGT
**Primers to generate IRK deletions**		
ΔJxKD; ΔKD	IRKcod_F	CACCATGTACAAAGCACTGATTTTTACAGTC
ΔKD	IRKmut_trunc_R	ATTTGTTCCAGTGCTGAAATC
ΔJxKD	IRKtrunc2_R	TACTGCTGATCTTGAAACCGT
**Primers to generate KOIN deletion**		
ΔJxKD	KOINcod_F	CACCATGATGCAGTCCATTTCCAGTT
ΔJxKD	KOINcod_trunc-JxK_R	GCTGTCTTCCCTCTTGTCC
**Primers to clone mSCARLETi**		
mSCARLET	mSCRLTi_p2p3_cod F	ATGGTGTCCAAGGGCGAA
	mSCRLTi_p2p3_cod R	TTACTTGTACAACTCGTCCATACC
**Primers used for KOIN qPCR**		
KOIN.1	KOIN V1_ex1/2_qF2	CTCCCAGGGTCAAGAAGTCA
	KOIN_ex2_qR2	TGCCTATCCGAGTTCAGGTC
KOIN.2 and KOIN.3	KOIN V2_ex1/2_qF2	GTGTTAAAGGTCAAGAAGTCAAGC
	KOIN_ex2_qR2	TGCCTATCCGAGTTCAGGTC
PP2A	PP2A_qF	TAACGTGGCCAAAATGATGC
	PP2A_qR	GTTCTCCACAACCGCTTGGT

Supplementary References

1. van Oostende-Triplet, C. *et al.* Vesicle dynamics during plant cell cytokinesis reveals distinct developmental phases. *Plant Physiol.* **174**, 1544–1558 (2017).
2. Richter, S. *et al.* Delivery of endocytosed proteins to the cell–division plane requires change of pathway from recycling to secretion. *Elife* **3**, e02131 (2014).
3. Kurepa, J., Karangwa, C., Duke, L. S. & Smalle, J. A. Arabidopsis sensitivity to protein synthesis inhibitors depends on 26S proteasome activity. *Plant Cell Rep.* **29**, 249–259 (2010).
4. Jásik, J. & Schmelzer, E. Internalized and Newly Synthesized Arabidopsis PIN-FORMED2 Pass through Brefeldin A Compartments: A New Insight into Intracellular Dynamics of the Protein by Using the Photoconvertible Fluorescence Protein Dendra2 as a Tag. *Mol. Plant* **7**, 1578–1581 (2014).
5. Li, R. *et al.* Different Endomembrane Trafficking Pathways Establish Apical and Basal Polarities. *Plant Cell* **29**, 90–108 (2017).
6. Zhang, C. *et al.* Endosidin2 targets conserved exocyst complex subunit EXO70 to inhibit exocytosis. *Proc. Natl. Acad. Sci.* **113**, E41 LP-E50 (2016).

7. Lešková, A. *et al.* Endosidin 2 accelerates PIN2 endocytosis and disturbs intracellular trafficking of PIN2, PIN3, and PIN4 but not of SYT1. *PLoS One* **15**, e0237448 (2020).
8. Wang, J., Cai, Y., Miao, Y., Lam, S. K. & Jiang, L. Wortmannin induces homotypic fusion of plant prevacuolar compartments*. *J. Exp. Bot.* **60**, 3075–3083 (2009).
9. Faculty, P., Academy, P. P. & Biology, C. The secretory vesicles tethering complex exocyst and the auxin transport polarization Ph . D . Thesis Mgr . Edita Janková Drdová Prague 2011 Supervisor : Consultant : 1–17
10. Renna, L. *et al.* TGNap1 is required for microtubule-dependent homeostasis of a subpopulation of the plant trans-Golgi network. *Nat. Commun.* **9**, 5313 (2018).
11. Geldner, Niko, Jiří Friml, York-Dieter Stierhof, Gerd Jürgens, and Klaus Palme. "Auxin transport inhibitors block PIN1 cycling and vesicle trafficking." *Nature* **413**, no. 6854: 425-428 (2001).
